# Systemic and Histopathological Effects of Bromelain-Based Enzymatic Debridement in Human Burns—A Systematic Review

**DOI:** 10.3390/medsci14060667

**Published:** 2026-10-13

**Authors:** Andrei-Nicolae Gologan, Mihai-Codrin Constantinescu, Dan-Cristian Moraru, Stefana Avadanei-Luca, Alexandru-Hristo Amarandei, Dimitrie-Stefan Gologan, Bianca-Beatrice Carausu, Delia-Gabriela Ciobanu-Apostol, Mihaela Pertea

**Affiliations:** 1 Grigore T. Popa University of Medicine and Pharmacy Iasi, 700115 Iași, Romania; andrei-nicolae.gologan@d.umfiasi.ro (A.-N.G.); mihai-codrin.constantinescu@umfiasi.ro (M.-C.C.); stefana_luca@umfiasi.ro (S.A.-L.); alexandru-hristo.amarandei@d.umfiasi.ro (A.-H.A.); mg-rom-33503@students.umfiasi.ro (D.-S.G.); biancabcarausu@gmail.com (B.-B.C.); delia.ciobanu@umfiasi.ro (D.-G.C.-A.); mihaela.pertea@umfiasi.ro (M.P.); 2 Department of Plastic Surgery and Reconstructive Microsurgery, Sf. Spiridon Emergency County Hospital, 700111 Iasi, Romania; 3 Department of Dermatology, Sf. Spiridon Emergency County Hospital, 700111 Iasi, Romania

**Keywords:** burns, enzymatic debridement, bromelain, NexoBrid^®^, histopathology, coagulation, inflammation, systematic review

## Abstract

**Background/Objectives:** Bromelain-based enzymatic debridement (NexoBrid^®^) is an established tissue-preserving alternative to tangential excision in deep partial-thickness burns. Its clinical efficacy is supported by randomised trials, but its systemic effects are inconsistently described and its effects at tissue level in humans are largely unknown. The objective was to synthesise the human evidence on both domains and to determine, as a prespecified endpoint, whether any study has evaluated them in the same patients. **Methods:** PubMed, Embase and Scopus were searched between 2016 and 2026 using two stream-specific strategies that shared their intervention and population blocks and differed only in their outcome block: Stream A, clinical outcomes reported with systemic biological parameters; Stream B, clinical outcomes reported with histopathological findings. Eligible studies were original human investigations published in the last ten years. Risk of bias was assessed with ROBINS-I and the JBI checklists, and the synthesis was narrative, following PRISMA 2020 and SWiM. **Results:** Of 559 records, twelve studies were included: ten in Stream A (970 patients) and two in Stream B (nine patients); no study identified by the present search met the criteria of both. C-reactive protein and procalcitonin were significantly higher after enzymatic than after surgical debridement throughout the first week, and, in the single study that analysed the treated area as a continuous exposure, the probability of a post-debridement coagulation alteration rose with the area treated (odds ratio 1.158 per percentage point of body surface). Early fever occurred in 14% of patients and predicted later wound infection. Both histological studies were purely qualitative: neither reported a thickness, a percentage or a cell count, and neither reported any laboratory value. **Conclusions:** Enzymatic debridement is associated with a measurable systemic response whose proposed mechanisms are all statements about tissue that has never been examined. The two literature fields describe the same process at two levels of observation, in different patients and at incompatible times. No validated instrument exists for quantifying the human burn biopsy after debridement.

## 1. Introduction

### 1.1. Epidemiological and Clinical Burden of Burn Injury

Burn injuries remain a major global health challenge and are among the leading causes of trauma-related morbidity and mortality worldwide. An estimated 180,000 deaths are caused by burns every year, the vast majority in low- and middle-income countries, and non-fatal burn injury is a leading cause of morbidity [1]. Beyond mortality, burns are associated with substantial long-term disability, functional impairment, prolonged rehabilitation and high healthcare costs, representing a considerable socioeconomic burden [2]. Burn severity is determined primarily by burn depth, the total body surface area (TBSA) involved, the aetiological context, patient age, anatomical location, associated trauma, comorbidities and the presence of inhalation injury—all of which directly influence treatment strategy and clinical outcome [2,3].

### 1.2. Eschar Removal and the Limits of Tangential Surgical Excision

For more than five decades, tangential surgical excision has remained the standard treatment for deep partial-thickness and full-thickness burns [4]. However, this technique relies on subjective intraoperative assessment of tissue viability and may result in unnecessary excision of viable dermis, particularly in deep partial-thickness burns, where viable and necrotic tissues coexist within the zone of stasis. Tangential excision is additionally associated with significant intraoperative blood loss, the need for general anaesthesia and, frequently, with an expansion of the donor-site burden through autologous skin grafting.

### 1.3. Bromelain-Based Enzymatic Debridement

Bromelain-based enzymatic debridement (NexoBrid^®^, MediWound Ltd., Yavne, Israel) has emerged as a selective, tissue-preserving alternative to conventional surgical excision and is currently used primarily in the management of deep partial-thickness and mixed-depth thermal burns [5]. NexoBrid^®^ consists of a concentrate of proteolytic enzymes enriched in bromelain, extracted from the stem of *Ananas comosus*. By selectively hydrolysing heat-denatured collagen and other necrotic extracellular matrix components while preserving viable dermis and the dermal microvascular network, enzymatic debridement enables a more accurate assessment of burn depth after eschar removal and may reduce the need for autologous skin grafting [6,7,8].

A multicentre randomised controlled trial and subsequent clinical studies have consistently demonstrated rapid and effective eschar removal, reduced blood loss, improved preservation of viable tissue and a decreased rate of surgical intervention in appropriately selected patients [9]. These data supported the incorporation of bromelain-based enzymatic debridement into contemporary European consensus recommendations and burn management guidelines [10], and its adoption is now reflected in large single-centre series [11].

### 1.4. The Systemic Biological Dimension: An Incompletely Characterised Effect

Although the clinical efficacy of bromelain-based enzymatic debridement is now well established, its biological effects remain incompletely characterised. Severe burn injury induces a complex systemic inflammatory response involving the activation of innate immunity, endothelial dysfunction, coagulation abnormalities and profound metabolic changes, all of which influence wound healing and patient outcomes [2]. Enzymatic debridement is superimposed on this response: it is applied to a large, non-intact tissue surface, it removes the eschar barrier within four hours, and bromelain itself has documented fibrinolytic and anti-aggregatory properties [12,13]. It is therefore biologically plausible that the procedure modifies systemic haemostatic, inflammatory and microbiological parameters.

This plausibility is supported by a converging body of clinical observations. Coagulation abnormalities following NexoBrid^®^ application—most consistently a transient prolongation of activated partial thromboplastin time and a decrease in fibrinogen—have been reported in cohort studies and case reports [14], including in minor burns [15], and have raised questions regarding the threshold for haemorrhagic risk. Independently, the abrupt removal of the eschar has been examined as a potential trigger for bacterial translocation, with studies addressing the incidence of bacteraemia after enzymatic debridement [16] and the evolution of the burn wound microbial profile under this treatment [17]. Safety analyses in patients with extensive burns exceeding 15% TBSA have specifically examined systemic tolerance, laboratory derangements and organ dysfunction [18].

### 1.5. The Tissue-Level Dimension: A Scarce and Non-Standardised Evidence Base

Histopathological assessment provides unique insight into extracellular matrix preservation, angiogenesis, inflammatory cell infiltration and dermal remodelling, allowing direct evaluation of tissue-level mechanisms that cannot be appreciated through clinical examination alone. It is, in principle, the only method capable of verifying the central claim made for bromelain-based enzymatic debridement—namely, selectivity.

The selectivity of the agent has been demonstrated in preclinical models, chiefly porcine comb-burn and mid-dermal burn models, in which preservation of the zone of stasis, retention of viable dermal thickness and accelerated re-epithelialization were quantified histologically [6,7,8]. In human burns, by contrast, the published histopathological evidence is limited to a small case series of eight patients assessed before and after debridement [19] and to a single case report describing pseudoeschar formation [20]. The histopathological changes following bromelain-based enzymatic debridement concluded that, after more than a decade of clinical use, human histological data derive from a total of nine patients, and identified the absence of prospective studies with systematic biopsies at standardised intervals and quantitative histomorphometric analysis as the principal translational gap [21].

Existing histological scoring systems were developed in murine or porcine wound-healing models, address spontaneous healing rather than debridement, and were not designed for the specific tissue findings produced by proteolytic escharolysis—homogenised upper dermis, residual necrosis, and pseudoeschar. As a consequence, the few available human descriptions are qualitative and are not comparable across studies. This defines the *histological stream* of the evidence and, at the same time, the methodological problem addressed in the present work.

### 1.6. The Missing Link Between the Two Dimensions

Studies have focused on clinical outcomes, systemic biochemical alterations or histopathological changes separately, while—to the best of our knowledge—none has attempted to relate the systemic biological response to the microscopic tissue response within the same patient population. The systemic markers that are routinely measured (inflammatory, coagulation, microbiological) and the tissue events that determine outcome (dermal preservation, re-epithelialization, burn conversion) are therefore described in parallel but never connected.

This disconnection is not merely a bibliographic observation. If a systemic biomarker profile were shown to correspond to a defined pattern of dermal preservation, it would provide a non-invasive surrogate for tissue viability and could inform, at the bedside, the decision between conservative management and grafting. The absence of any such study is, in itself, the principal finding that the present review sets out to document and to substantiate.

### 1.7. Aim, Review Question and Objectives

The aim of the present systematic review is to critically synthesise the available human evidence regarding the effects of bromelain-based enzymatic debridement on clinical outcomes, systemic biological alterations and histopathological changes following thermal burns, and to determine whether any published study has integrated the biological and the histopathological dimensions within the same cohort.

By integrating these complementary domains, this review aims to provide a translational perspective on burn wound healing after enzymatic debridement, to identify current knowledge gaps and to define priorities for future prospective clinical research—including the instrument required to address them.

## 2. Materials and Methods

### 2.1. Protocol, Registration and Reporting Standards

This systematic review was designed, conducted and reported in accordance with the Preferred Reporting Items for Systematic Reviews and Meta-Analyses (PRISMA 2020) statement [22]. Because a quantitative synthesis was not planned, the narrative synthesis is additionally reported according to the Synthesis Without Meta-analysis (SWiM) guideline [23]. The completed PRISMA 2020 checklist and the SWiM checklist are provided as Supplementary Material. The systematic review protocol was registered prospectively in the International Prospective Register of Systematic Reviews (PROSPERO), registration number CRD420261492222.

### 2.2. Information Sources and Search Strategy

#### 2.2.1. Databases and Search Execution

Electronic searches were performed in PubMed/MEDLINE, Embase (Elsevier) and Scopus, from database inception to 26 August 2026. Two stream-specific strategies were executed in each database, giving six searches in total. Each strategy combined three concept blocks with the Boolean operator AND: (i) the intervention block (NexoBrid^®^, bromelain-based enzymatic debridement, enzymatic debridement, enzymatic necrosectomy); (ii) the population block (burn, thermal injury, burn wound); and (iii) an outcome block that differed between the two streams. Within each block, terms were combined with OR, and controlled vocabulary (Emtree in Embase) was used together with free-text terms in title-and-abstract fields. Truncation was applied to capture morphological variants.

The outcome block of Stream A comprised clinical, laboratory, inflammatory, coagulation and infection descriptors (clinical, outcome, healing, graft, epithelialization, infection, bacteremia, bleeding, blood loss, biomarker*, laboratory, inflammatory, inflammation, coagulation, CRP, C-reactive protein, procalcitonin, leukocyte*, platelet*, fibrinogen, INR, aPTT, D-dimer, albumin, haemoglobin). The outcome block of Stream B comprised tissue-level and morphological descriptors (clinical, outcome, healing, graft, epithelialization, infection, scar, histolog, histopatholog, biopsy, immunohistochem, immunostain, tissue remodelling, remodelling, collagen, elastin, extracellular matrix, angiogenesis, microvascular, vascular, CD31, CD34, VEGF, Ki-67, MMP*, pseudoeschar). The complete, verbatim, database-specific search strings, together with the number of records retrieved by each, are reproduced in Supplementary Table S1.

Reference lists of all included articles and of the relevant systematic reviews identified during screening were hand-searched for additional eligible studies (backward citation tracking). No additional records fulfilling the eligibility criteria were identified by this route. No unpublished or grey literature sources were searched, and this is declared as a limitation.

#### 2.2.2. Iterative Refinement of the Strategy and Declared Protocol Deviation

The search strategy was developed iteratively, in order to maximise retrieval of clinically relevant studies while preserving methodological rigour. The initial, highly restrictive strategies—which required the simultaneous presence of enzymatic debridement, burn, biochemical and histopathological concepts within the same record—returned a small number of records, none of which fulfilled all predefined eligibility criteria at full-text assessment.

This constitutes a deviation from the originally conceived design and is declared as such. Three points define its methodological status: the deviation concerned the search architecture and not the review question, which remained unchanged; it was decided and documented before the final screening and selection process; and it was motivated by an empirical finding—the scarcity of studies addressing all three domains—that is itself reported as a principal result of this review. The refinement therefore did not weaken the original objective but converted it from an inclusion criterion into a testable endpoint.

### 2.3. Eligibility Criteria

The ten-year time frame requires explicit justification. Bromelain-based enzymatic debridement received marketing authorisation from the European Medicines Agency in December 2012 and entered routine clinical use from 2013; approval by the United States Food and Drug Administration followed only in December 2022, so that the entire clinical literature available to this review is of European origin and the European authorisation date is the relevant anchor. The authors agreed by consensus, at the protocol stage, that a minimum of approximately four years must elapse from marketing authorisation before a clinical literature usable in a systematic review accumulates—a period covering first clinical adoption, the completion of the first prospective series and their passage through peer review. Applied to a December 2012 authorisation, this rule yields 2016.

The eligibility criteria are summarised in Table 1.

Two design-related decisions require explicit statement. First, case reports and small case series were retained rather than excluded, because in Stream B they constitute the entirety of the available human evidence; their design is, however, formally accounted for in the risk-of-bias assessment, and they are never presented as equivalent to cohort data. Second, preclinical (porcine and murine) studies and in vitro investigations were excluded from the synthesis but were retrieved and read separately; they are used exclusively in the Discussion, to support the mechanistic interpretation of the human findings. This distinction—excluded from the evidence base, retained as interpretative and methodological support—is maintained throughout the manuscript.

### 2.4. Study Selection and Deduplication

**Figure 1:**
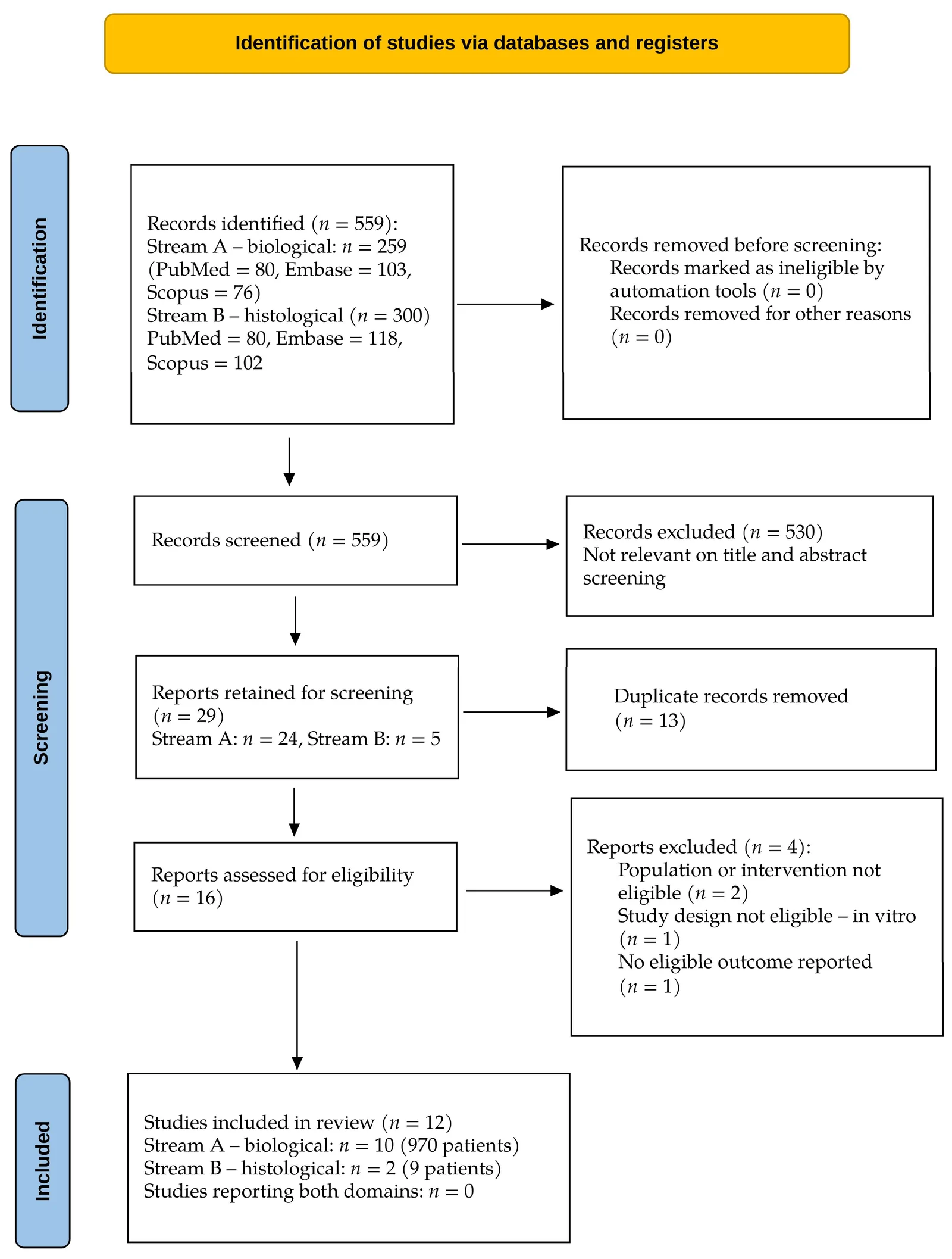
PRISMA 2020 flow diagram.

All records were exported to a reference manager, where deduplication of the records retained after title-and-abstract screening was performed in two stages: automated matching on digital object identifier, title and first author, followed by manual verification of every candidate duplicate pair. Deduplication was applied both across databases within a stream and across the two streams, since the intervention and population blocks were shared and a substantial overlap was expected by design. Thirteen duplicate records were removed. Titles and abstracts were screened independently by two reviewers (A-N.G. and M-C.C.) against the eligibility criteria. Full texts of all potentially relevant records were retrieved and assessed independently by the same two reviewers. Disagreements were resolved by discussion and, where consensus was not reached, by a third reviewer (M.P.). No automation tool was used at any stage of screening or data extraction. Reasons for exclusion at the full-text stage were documented for every record and are reported in the PRISMA flow diagram (Figure 1) and in Supplementary Table S2.

### 2.5. Data Extraction

Data were extracted by one reviewer (A-N.G.) using a standardised, pre-piloted data collection form and verified independently by a second reviewer (M-C.C.). The form was piloted on three studies and revised before full application. The following items were extracted for each included study: first author, year, country and centre; study design and setting; sample size; patient demographics (age, sex, comorbidities); burn characteristics (aetiology, TBSA, depth, anatomical distribution, inhalation injury); the enzymatic debridement protocol (indication, time from injury to application, TBSA treated per session, number of applications, anaesthesia and analgesia, soaking and dressing regimen); comparator, where present; clinical outcomes (time to complete debridement, need for and extent of autografting, time to complete wound closure, length of hospital stay, scar assessment, mortality); systemic biological parameters (analyte, sampling time points, absolute values or reported change, statistical significance); histopathological findings (biopsy site and timing, fixation and staining, descriptors used, any scoring instrument applied, findings before and after debridement); adverse events; follow-up duration; funding and conflicts of interest; and the authors’ principal conclusions.

Where data were reported only graphically, or where relevant subgroup data were not extractable, this was recorded as such; no attempt was made to impute values, and study authors were not contacted. Data extracted from case reports were handled at the individual-patient level.

### 2.6. Risk-of-Bias Assessment

Risk of bias was assessed independently by two reviewers (A-N.G. and M-C.C.), using design-appropriate instruments. Non-randomised comparative and cohort studies were appraised with the ROBINS-I tool across its seven domains—confounding, participant selection, classification of interventions, deviations from intended interventions, missing data, measurement of outcomes, and selection of the reported result—with an overall judgement of low, moderate, serious or critical risk of bias [24]. Case series and case reports were appraised with the corresponding Joanna Briggs Institute critical appraisal checklists [25]. Disagreements were resolved by consensus, with recourse to a third reviewer (M.P.) where required.

For Stream A, two domains were specified a priori as the dominant sources of bias: confounding by burn severity and by concurrent surgical intervention, and the selective reporting of laboratory parameters, since biomarker panels and sampling time points were investigator-defined and not protocol-driven in any retrieved study. For Stream B, the small number of participants, the absence of an untreated comparator biopsy and the qualitative nature of the histological reporting were specified as the dominant limitations. Risk of bias was not used as an exclusion criterion; it informs the weight given to each study in the narrative synthesis and is reported alongside every finding.

### 2.7. Data Synthesis

Because of substantial anticipated heterogeneity in study design, patient populations, enzymatic debridement protocols, outcome definitions, laboratory panels, sampling time points and histological assessment methods, a quantitative meta-analysis was not considered appropriate and was not performed. Results were therefore synthesised narratively and reported according to SWiM [23].

The synthesis was structured according to the two evidence streams and, within each stream, according to predefined outcome groups: for Stream A, (i) coagulation and haemostasis, (ii) infection, bacteraemia and the wound microbiological profile, (iii) inflammatory and biochemical markers, and (iv) clinical outcomes and safety in extensive burns; for Stream B, (i) pre-debridement histology, (ii) post-debridement histology and dermal preservation, and (iii) enzymatic debridement–specific findings, including pseudoeschar. Studies were grouped by outcome rather than by design, and within each group they are presented in order of decreasing methodological robustness. Vote counting based on statistical significance was avoided; direction and magnitude of effect are reported with their reported uncertainty, and consistency across studies is described narratively. In the narrative text the studies are presented in this order; in the extraction and risk-of-bias tables they are listed chronologically, by year of publication.

Finally, the findings of the two streams were juxtaposed in an integrative section, in which each systemic biological signal is examined against the tissue-level process that could plausibly generate it, and the evidential status of that link—demonstrated, inferred, or absent—is stated explicitly. This section operationalizes the primary a priori endpoint of the review.

No formal assessment of the certainty of the body of evidence was undertaken. Because no estimate was pooled, and because the evidence base consists of non-randomised studies, single-study findings and case reports, a structured framework such as GRADE would in our judgement have returned a rating of low or very low certainty for every outcome group without discriminating among them. The omission is declared here and in the Limitations, and it carries one consequence that is stated once and applies throughout the manuscript: no statement in this review constitutes a graded judgement of certainty. Where the direction, magnitude or consistency of an effect is described, it refers to what the included studies report, and not to confidence that the reported effect is correctly estimated.

## 3. Results

### 3.1. Study Selection

The two stream-specific search strategies retrieved 559 records: 259 in the biological stream (PubMed 80, Embase 103, Scopus 76) and 300 in the histological stream (PubMed 80, Embase 118, Scopus 102). Title-and-abstract screening excluded 530 records, leaving 29. Removal of duplicates between databases and between the two streams eliminated 13 records, and 16 full texts were assessed for eligibility.

Four articles were excluded at the full-text stage: two because enzymatic debridement was not the studied intervention in an eligible population, one on design grounds as an in vitro laboratory study, and one because it reported clinical and scar outcomes without any systemic biological parameter and without histopathology, meeting the criteria of neither stream. All four are listed with their reasons for exclusion in Supplementary Table S2; like the preclinical studies, they are retained for the Discussion.

Twelve studies were included in the qualitative synthesis: ten in the biological stream and two in the histological stream. No study identified by the present search met the criteria of both streams. Backward citation tracking of the included articles and of the systematic reviews identified during screening yielded no additional eligible study. Full texts were obtained and read for all twelve included studies, so every extraction reported below is based on the published article rather than on its abstract. Study selection is summarised in the PRISMA 2020 flow diagram (Figure 1).

### 3.2. Characteristics of the Included Studies

The general characteristics of the studies are presented in Table 2.

### 3.3. Stream A—Extraction Tables

No two studies measured the same set of analytes at the same time points. The panels ranged from three parameters at five time points to more than twenty parameters daily for a week, and two studies measured no blood analyte at all. The full list of parameters and sampling schedules is given in Supplementary Table S3; this heterogeneity is the structural reason why quantitative pooling was not possible.

#### 3.3.1. Coagulation and Haemostasis Findings

Seven studies bear on haemostasis. The four group-level comparisons against surgical debridement or against a smaller treated area find no clinically relevant derangement. Bulla [11], the only study to examine the treated area as a continuous exposure, finds a clear dose–response relationship with both coagulation alteration and transfusion. The two case-level reports capture what group means conceal: a transient, fully reversible prothrombin prolongation (Martín [26]) and a life-threatening haemorrhage of anatomical rather than haemostatic origin (Hasham [27], case 2) (Table 3).

#### 3.3.2. Infection, Bacteraemia and Wound Microbiological Profile

The two adjusted analyses diverge sharply. Deplazes [16] (*n* = 269, 83 exposed) finds no association between bromelain and bacteraemia, whereas Kaita [29] (*n* = 17, seven exposed) reports a large protective odds ratio whose confidence interval reaches 0.96—statistically significant but extremely imprecise. Bulla [11] contributes the only prospective-in-record observation linking a systemic sign to a later outcome: early fever, present in 14%, predicts subsequent wound infection with an odds ratio of 2.5. Sharaf [17], the only study designed specifically to test the microbiological question, reports no statistical comparison at all, and its two cohorts differ five-fold in mean TBSA.

All results are available in Table 4.

#### 3.3.3. Inflammatory, Biochemical and Haematological Findings

The inflammatory findings are the most informative and the most contradictory in the review. Pfister [14], comparing enzymatic against surgical debridement in 132 patients, finds CRP and procalcitonin significantly and persistently higher after enzymatic debridement across seven days. Breidung [15], in a within-patient design in burns under 10% TBSA, finds a highly significant CRP rise within 24 h. Hofmaenner [18] finds no difference—but its comparison is between two enzymatically debrided groups, so it tests the effect of treated area, not the effect of the procedure, and its only significant CRP difference is a baseline imbalance. Read together, the three are compatible: enzymatic debridement is followed by a measurable acute-phase response that does not scale with the area treated (Table 5).

#### 3.3.4. Clinical Outcomes

Only two studies report a statistically significant between-group difference for a clinical endpoint (Kaita [29], time to complete debridement; Hofmaenner [18], survival). Bulla [11] is the only study relating the post-debridement assessment itself to outcome, and shows that the post-ED grade predicts both the need for surgery and later hypertrophy far better than the clinical assessment made on admission (Table 6).

### 3.4. Stream B—Histopathological Extraction

The complete human histopathological evidence base for bromelain-based enzymatic debridement comprises nine patients. Both studies are qualitative and neither reports a quantitative histological measurement or any systemic parameter. Two features of the Miura specimen [20] deserve note: it was taken across the wound margin so that intact skin and burned tissue appear in the same section, providing an internal reference, and it was stained with four techniques rather than one, which is what permitted the composition of the pseudoeschar to be established (Table 7).

### 3.5. Risk of Bias in the Included Studies

Risk-of-bias assessment was performed after retrieval of all twelve full texts. Residual data gaps in the source studies are itemised in Supplementary Table S4. No study was excluded on the basis of its risk of bias. Two points warrant emphasis: Sharaf 2022 [17], the only study designed specifically to answer the microbiological question, carries a critical risk of bias and reports no statistical comparison, and Hofmaenner 2021 [18], the study most often cited as evidence that enzymatic debridement provokes no systemic inflammatory response, compares two enzymatically debrided groups rather than enzymatic against surgical debridement, and carries manufacturer-related conflicts of interest (Table 8).

A narrative sensitivity assessment was performed, since no estimate was pooled and a quantitative sensitivity analysis is therefore not available. Excluding the study at critical risk of bias [17] and the four studies at serious risk does not affect the primary endpoint, because the intersection of the two streams is empty irrespective of which studies are retained. The inflammatory signal is likewise unaffected, resting on Pfister 2023 [14], at moderate risk, and on Breidung 2025 [15]. The coagulation dose–response rests on a single study [11] and does not survive its removal, which is stated here as such. The microbiological question becomes entirely unresolved: excluding Sharaf 2022 [17] leaves only the well-adjusted null cohort [16] and a seven-patient estimate whose confidence interval reaches 0.96 [29]. This assessment indicates which findings persist when the least reliable studies are removed; it is not, and is not presented as, a formal rating of the certainty of the evidence.

### 3.6. Integration of the Two Evidence Streams

#### 3.6.1. The Intersection of the Two Streams Is Empty

The primary a priori endpoint of this review was the number of studies evaluating both the systemic biological and the histopathological dimension within the same patient population. That number is zero. Table 9 cross-tabulates the three outcome domains across the twelve included studies.

The finding is not an artefact of the search strategy. The two streams were searched with deliberately overlapping intervention and population blocks, differing only in the outcome block, so any study reporting both a biological and a histological outcome would have been retrieved twice and assigned to both streams. Thirteen records were in fact duplicated across the two searches; none of them reported both outcome domains. The result is corroborated externally: a systematic review of the histopathological literature published independently in 2026, which searched five databases without a date limit and included six preclinical and two clinical studies, reports an endpoint inventory that is entirely morphological and identifies no study, in any species, correlating systemic biological parameters with histopathological findings after bromelain-based enzymatic debridement [21].

#### 3.6.2. What the Two Streams Describe, and Where They Fail to Meet

The two bodies of evidence are not merely separate. They describe the same biological process at two levels of observation, in different patients, with no connecting measurement. Four juxtapositions make this concrete, and in the first of them the authors of the biological study state the missing link themselves.

Inflammation—the most consistent signal, and an explicit unanswered question. Pfister et al. [14], comparing 66 enzymatically against 66 surgically debrided patients, found C-reactive protein and procalcitonin significantly and persistently higher after enzymatic debridement across the whole first week (both *p* < 0.01; median CRP on day 5, 213 vs. 137 mg/L). They attribute this to “an increased inflammatory local tissue reaction” producing a greater systemic response, and go one step further: they propose that this heightened inflammation could account for the elevated factor V they also observed, and write that the correlation “remains to be investigated”. Breidung et al. [15] independently found a highly significant CRP rise within 24 h in burns under 10% TBSA. The corresponding tissue observations exist: Di Lonardo et al. [19] describe an abundant inflammatory infiltrate at debridement, denser in the deep dermis than superficially, and Miura et al. [20] describe inflammatory cells spiralling upward from the lower dermis through the ulcer layer. The systemic measurement and the tissue measurement each exist, in the same disease, at overlapping times—and never in the same patient. The proposed “increased local tissue reaction” that explains the CRP rise has never been measured in tissue.

Haemostasis and the vascular bed: Bulla et al. [11] found, in 300 patients, that the probability of a post-debridement coagulation alteration rose with the area treated, with an odds ratio of 1.158 per percentage point of TBSA (95% CI 1.103–1.224), and the same dose–response held for transfusion. They state plainly that their design cannot distinguish a direct effect of bromelain on the coagulation cascade from consumption of clotting factors through blood loss. The histological observations that would discriminate between the two are precisely those already described: Di Lonardo et al. [19] report marked vascular congestion with blood extravasation through disrupted capillaries in the ecchymotic zones, and Miura et al. [20] report that capillaries in the superficial reticular dermis were absent while those in the deeper dermis remained intact. A dose-dependent systemic coagulation change and a dose-dependent area of disrupted dermal microvasculature are the same hypothesis stated twice, once from the blood and once from the section. No study has stated it in both.

Fever, infection and the state of the wound bed: Bulla et al. [11] observed early post-debridement fever in 14% of patients, more frequent in younger patients—which they read as inflammatory rather than infective—and showed that this fever predicted later wound infection with an odds ratio of 2.5 (95% CI 1.09–5.49). Deplazes et al. [16] found a median time to first bacteraemia of six days. Miura et al. [20] observed pseudoeschar formation on day 6 and recorded, in the same sentence-length clinical statement, that there were no signs of systemic infection. That single observation—the state of the wound bed and the systemic status of the patient noted together—is the closest the entire literature comes to an integrative datum, and it is one patient, described qualitatively, with no laboratory value attached.

Tissue decompression: Kaita et al. [29] measured intratissue pressures falling from 81 to 12 mmHg in an upper arm and from 52 to 20 mmHg in a hand within four hours of application: a direct, quantified tissue-level effect measured with a pressure transducer and never with a microscope. No histological correlate of this decompression has been described in any human study.

#### 3.6.3. Why the Gap Exists

Three structural reasons account for the absence, and each of them is remediable.

1. Disciplinary separation: The biological stream originates almost entirely from intensive care and burn surgery departments; the histological stream from pathology. Of the ten Stream A studies, none involved a pathologist as an author. Of the two Stream B studies, neither reports a single laboratory value—although Miura et al. [20] did include a pathologist as a co-author, which is what made the four-stain analysis possible.
2. The invasiveness of the measurement: Serial biopsy of a burn wound is not part of routine care, whereas serial blood sampling is. The biological stream therefore accumulated retrospectively from data collected for clinical purposes, reaching 970 patients in ten studies, while the histological stream required deliberate prospective sampling and has accumulated nine patients in thirteen years of clinical use.
3. The absence of a quantitative histological endpoint: Correlation requires two quantities. Both human histological studies are purely qualitative: neither reports a single thickness, percentage or cell count. Even if a study had measured C-reactive protein and taken a biopsy in the same patient, there would have been no instrument with which to convert the biopsy into a number that could be correlated against it. Until a quantitative instrument exists for the human burn biopsy after enzymatic debridement, no correlation of this kind is possible in principle, whatever the study design.

Two observations suggest the gap is narrower in practice than it appears in print. The Miura specimen [20] was an incisional biopsy taken across the wound margin, so that intact skin and burned tissue appeared in the same section—a construction that provides an internal intact reference, and evidence that such sampling is feasible in an acute burn patient. Furthermore, Pfister et al. [14] collected, in the same cohort and on the same days, both the coagulation panel and the inflammatory panel that a histological correlation would require; only the biopsy was missing.

#### 3.6.4. Temporal Non-Alignment

A fourth obstacle is more easily overlooked and equally consequential. The two streams sample at incompatible times. Biological sampling clusters in the first hours to days: within 24 h either side of application (Breidung [15]), from 24 h before to 72 h after (Hofmaenner [18]), from the day before to three days after (Capitelli-McMahon [28]), and daily from day 1 to day 7 (Pfister [14]). Histological sampling occurred at debridement itself, with two specimens at 24 h in one patient (Di Lonardo [19]), and on day 10 (Miura [20]).

The consequence is precise. Di Lonardo’s [19] 24 h biopsies fall inside the sampling window of four of the biological studies, but were taken in a different country, in a different patient, with no laboratory value recorded. Miura’s [20] day 10 biopsy falls outside the biochemical and haematological sampling window of every Stream A study: none measured an inflammatory, biochemical or coagulation parameter on day 10, and the only observations extending that far are microbiological surveillance [16,17] and two isolated late clotting profiles in a case series [27]. The pseudoeschar, the one tissue phenomenon specific to this intervention, forms and is examined in a period during which the systemic response has never been characterised. Any future study intending to relate the two must specify shared time points in advance rather than hope to align data collected for other purposes, must extend biological sampling well beyond the first week, and must extend the histological schedule beyond the moment of debridement itself.

#### 3.6.5. Summary of the Primary Finding

The asymmetry between the two streams is itself part of the finding. The biological stream is large, quantitative and internally contradictory: it reports a persistent acute-phase response after enzymatic debridement (Pfister [14], Breidung [15]) alongside a study finding none (Hofmaenner [18]), and a dose–response for coagulation alteration reported by one study (Bulla [11]) alongside four studies finding no group-level derangement. The histological stream is minute, entirely qualitative, and internally consistent only because two studies cannot disagree. Neither stream can resolve its own contradictions from within, because the tissue explanation proposed for every biological finding—increased local inflammatory reaction, factor consumption in a disrupted microvasculature, wound-bed status governing infection—is never measured, and the tissue findings are never anchored to a systemic variable.

This is the principal result of the present review, and it defines the design requirement for the prospective study that follows from it: the same patients, quantitative histology at fixed time points, a systemic panel sampled on the same schedule, and biological sampling extended beyond the first week to cover the period in which the pseudoeschar forms.

## 4. Discussion

The review set out to determine what is known about the biological and the tissue-level effects of bromelain-based enzymatic debridement in human burns, and whether any study has examined the two together. Twelve studies met the eligibility criteria: ten reporting systemic biological parameters in 970 patients, and two reporting histopathology in nine. None reported both. The clinical efficacy of the intervention is established by two randomised controlled trials [9,30] and endorsed by European consensus [31]; what remains unresolved is the biology that underlies it.

### 4.1. A Measurable Systemic Response of Uncertain Origin

Seven studies bear on haemostasis. Four group-level comparisons found no clinically relevant derangement: no difference in INR, aPTT, fibrinogen, factor XIII or platelets against surgical debridement [14]; none in prothrombin time, aPTT or platelets against matched surgical controls, with no blood product required in the enzymatically debrided group [28]; no bleeding event and no transfusion difference between smaller and larger treated areas [18]; and only a modest fall in prothrombin ratio, from 82.0 ± 13.3 to 76.8 ± 14.9, in burns under 10% total body surface area [15]. The one study that treated the debrided area as a continuous exposure rather than a group label detected a signal the group-level comparisons could not: the probability of a post-debridement coagulation alteration rose with the extent treated, odds ratio 1.158 per percentage point of body surface (95% CI 1.103–1.224), with the same gradient for transfusion [11]. A dose–response of this kind is not readily explained by confounding by burn severity alone, although a single non-randomised study cannot exclude it, and the authors state that their design cannot separate a pharmacological effect from consumption of clotting factors through blood loss [11].

That distinction can be narrowed, though not settled, from pharmacological data. Contrary to the assumption that a topically applied enzyme remains local, the pharmacokinetic data of the summary of product characteristics report systemic serum exposure in every patient studied, with a median time to peak of 4 h, a mean peak concentration of 200 ± 184 ng/mL, and a significant correlation between exposure and both dose and treated body surface area in adults [32]. Systemic exposure therefore shares the same dose axis as the coagulation signal. Two observations point the same way: a juvenile porcine toxicology study reported dose-related prolongation of prothrombin and partial thromboplastin times with a fall in fibrinogen, fully reversible by 48 h [32], and the one human case of marked prothrombin prolongation normalised on precisely that schedule [26].

The counter-arguments are equally concrete. Roughly half of the absorbed enzyme binds α2-macroglobulin and α1-antichymotrypsin, protease-scavenging antiproteases whose binding neutralises activity against large substrates such as coagulation factors [32]. The in vitro anticoagulant effects of bromelain—prothrombin time prolonged by 47%, aPTT by 20%, ADP-induced platelet aggregation inhibited by 19%—required 0.4 U/mL added directly to whole blood [12]; those units cannot be converted to the ng/mL of the pharmacokinetic data without the specific activity of the preparation, so the comparison establishes plausibility, not equivalence [13]. In the pooled safety dataset of the two pivotal trials, the only reports of coagulopathy and of haemorrhagic anaemia occurred in the standard-of-care arm [33]. Furthermore, the patient in whom the most severe haemorrhage occurred bled from a breached venous varicosity within the treated field, with a normal clotting profile on admission and no reported haemostatic derangement—an anatomical mechanism, not a haemostatic one [27].

The competing explanations therefore reduce to one tissue-level question: how much dermal microvasculature is disrupted, and over how large an area. That question was answered, qualitatively, by both human histological studies—marked vascular congestion with extravasation through disrupted capillaries in one [19], and absence of capillaries in the superficial reticular dermis with intact deeper vessels in the other [20]—in patients from whom no coagulation parameter was recorded.

The inflammatory data show the same structure. Against surgical debridement, C-reactive protein and procalcitonin were significantly higher after enzymatic debridement throughout the first week, with median CRP of 213 against 137 mg/L on day 5 [14]; in a within-patient design confined to burns under 10% TBSA, CRP rose from 4.8 ± 5.8 to 10.8 ± 7.2 mg/dL within 24 h [15]. The one study reporting no inflammatory difference compared two enzymatically debrided groups against each other rather than against surgery, and its only significant CRP difference was a baseline imbalance present before treatment [18]; it therefore tests the effect of treated area, not the effect of the procedure. Read together, the three are compatible with an acute-phase response that follows enzymatic debridement but does not scale with the area treated. Notably, the authors of the largest comparison propose that this heightened inflammation may itself explain the factor V elevation they observed, and state that the correlation remains to be investigated [14]—a hypothesis about tissue, advanced from the blood, and never tested in tissue.

### 4.2. Fever and Infection: A Systemic Sign of Wound-Bed Origin

Early post-debridement fever occurred in 14% of a 300-patient series [11] and fever-related adverse events in 15.3% of enzymatically treated patients against 12% of controls in the pooled randomised data [33]—a concordance across very different designs, which makes an artefact of a single design less likely without establishing the frequency with precision. Its interpretation is not: fever was more common in younger patients, favouring an inflammatory rather than an infective origin, yet it predicted later wound infection with an odds ratio of 2.5 (95% CI 1.09–5.49) [11]. Whether the debrided wound bed is more or less susceptible to colonisation is equally unresolved. A well-adjusted cohort found no association with bacteraemia (subdistribution hazard ratio 0.79, 95% CI 0.42–1.48) [16]; the study designed specifically to test the microbiological question reported no statistical comparison at all, from cohorts differing five-fold in mean burn size [17]; and the only study reporting a protective effect did so from seven exposed patients, with a confidence interval reaching 0.96 [29]. An in vitro laboratory study, excluded from the synthesis on design grounds but informative here, found no zones of inhibition around the agent after 18 h of incubation, suggesting that any effect on the wound flora derives from selective removal of the eschar rather than from direct antimicrobial action [34]. The determinants proposed for all of these—dermal preservation, adnexal viability, whether pseudoeschar acts as a barrier or as a substrate—are tissue properties, and none has been measured.

### 4.3. Tissue Evidence: Robust in Swine, Effectively Absent in Humans

The selectivity that justifies the intervention was established histologically in porcine models, consistently across them. In a porcine comb-burn model, enzymatic debridement left partial-thickness rather than full-thickness necrosis in two-thirds of the interspaces representing the zone of stasis, against full-thickness necrosis in all control interspaces (*p* = 0.05) [7]. In deep partial-thickness porcine burns, mean re-epithelialization at day 7 was 47.6 ± 3.2% against 0% in controls (*p* = 0.001), with higher Ki-67 expression in debrided wounds [35]; in mid-dermal porcine burns, complete re-epithelialization occurred at 7.4 ± 0.8 against 9.1 ± 2.1 days [8]. Selective dissolution of eschar without damage to underlying dermis, donor sites or normal skin was demonstrated directly [6], and the effectiveness of debridement was shown to depend on the denaturation status of the collagen it encounters—a tissue property, not a clinical one [36].

None of this has been reproduced in humans. The two pivotal randomised trials, together enrolling several hundred patients, assessed burn depth by clinical evaluation and photography and took no biopsies [9,30,33]; a systematic review of 34 clinical studies contains no histopathological study and does not report fever as an outcome [37]; and a systematic review devoted specifically to the histopathology of enzymatic debridement concluded that human data derive from nine patients in more than a decade of use, that neither study employed immunohistochemistry, and that quantitative histomorphometry has never been performed [21]. The asymmetry is stark: the mechanism has been shown in swine, the efficacy in humans, and the two have never been shown in the same species.

Pseudoeschar illustrates the cost. Consensus regards it as part of healing rather than therapeutic failure, yet also states that once established it makes re-evaluation of the wound bed for grafting technically impossible [31]. Its only histological characterisation comes from a single patient, in whom it proved to consist of collagen and elastic fibres extruded upward from the lower dermis through the ulcer layer, with negative Alcian blue and periodic acid–Schiff excluding mucopolysaccharides and glycans [20]. That analysis required four stains. The authors themselves note that silver sulfadiazine, applied daily from day 6 and independently capable of inducing a pseudoeschar, cannot be excluded as a contributor [20]. One patient, one time point, and one confounder, for the tissue phenomenon that most distinguishes this intervention from surgical excision.

### 4.4. Where the Two Streams Should Meet: The Dermal Step

One observation offers an unusually tractable point of contact. In 300 patients, a visible step in the post-debridement wound bed—a transition between areas where the dermis is intact and where it is lost—raised the odds of requiring surgery 8.98-fold (95% CI 3.85–22.39) and the odds of later hypertrophy 3.67-fold (95% CI 1.44–10.11), while the depth assessed clinically on admission predicted neither [11]. Independently, in a study of 102 laser-Doppler-confirmed deep areas, dermal step-off in the wound bed emerged as an independent predictor of the need for grafting (*p* < 0.001), alongside visible and translucent fat lobules and visible or coagulated vessels [38]. This involved two centres, two designs, and one macroscopic sign that predicts both the operative decision and the eventual scar. The clinical stake of that decision is considerable: in a two-arm comparison of 30 patients, split-thickness grafting was required in 33% of those treated enzymatically against 80% of the surgically debrided controls, and was avoided altogether in 47% of the enzymatic group [39].

What that sign is, histologically, has never been examined. By its own description it marks the boundary between preserved and lost dermis—the transition zone, where Jackson’s zone of stasis is decided [3], and the region both human histological studies happened to sample: one through elliptical specimens deliberately spanning macroscopically different zones [19], the other through an incisional specimen taken across the wound margin so that intact and burned skin appeared in one section [20]. A quantified measure of residual dermal thickness at that boundary, related on one side to the macroscopic step and on the other to systemic parameters from the same patient, would connect all three domains in a single specimen. The technique exists; it has never been applied with a systemic panel attached.

### 4.5. Implications for Practice

Three implications follow, two of which define questions for prospective evaluation rather than changes in current practice. First, the European consensus requires coagulopathy to be corrected before application and coagulation to be checked peri-procedurally, but defines no post-application monitoring, no parameters and no timing [31]. Given a demonstrated dose–response between treated area and coagulation alteration [11], and given that in that same series no post-debridement coagulation test was performed at all in 74 of 300 patients, whether a defined post-application coagulation check in patients treated over larger areas reduces unrecognised coagulopathy or transfusion requirement is a question that merits prospective evaluation. Second, early fever occurs in roughly one in seven patients, is reproducible across designs [11,33] and predicts later wound infection [11]; whether a defined response—blood cultures and wound surveillance—improves the detection of subsequent wound infection should be tested prospectively rather than assumed. Third, the assessment made immediately after debridement predicts both the need for surgery and the eventual scar substantially better than the assessment made on admission [11], and specific wound-bed signs are reproducible between experienced and less-experienced users [38]; this supports the consensus emphasis on structured evaluation at the moment of debridement [31], before pseudoeschar makes it impossible.

### 4.6. Comparison with Existing Syntheses

Two syntheses address adjacent ground. A systematic review of 34 clinical studies established the safety and efficacy profile of the intervention, but includes no histopathological study, does not report fever as an outcome, and performs no quantitative pooling [37]. A systematic review published in 2026 addresses the histopathology directly, but includes six preclinical and two clinical studies and reports an endpoint inventory that is entirely morphological [21]. The present review differs in searching both domains with deliberately overlapping strategies that differ only in their outcome block, so that a study reporting both would necessarily have been retrieved twice—and in reporting the size of their intersection as a prespecified endpoint rather than as an incidental observation.

## 5. Limitations

The search architecture was restructured into two streams after preliminary searches retrieved no study meeting all three outcome domains. The change was decided and documented before final screening, concerned the search architecture rather than the review question, and converted the original objective from an inclusion criterion into a testable endpoint; it is nonetheless a deviation from the design as first conceived and is declared as such.

We searched three databases, restricted eligibility to English-language publications of the last ten years, and did not search grey literature, trial registries or conference proceedings, nor did we contact authors for unpublished data. A negative finding is more vulnerable to these restrictions than a positive one, since a single unretrieved study would overturn it. Two considerations limit that risk: the two streams shared their intervention and population blocks, so any study reporting both outcome domains would have been retrieved by both searches; and an independently conducted systematic review, which searched five databases without a date limit, reports no such study either [21]. The ten-year limit is unlikely to have excluded relevant work: the agent received European marketing authorisation in December 2012, entered routine clinical use from 2013, and was approved in the United States only in December 2022. The limit was set by author consensus at approximately four years after marketing authorisation, the interval required for a clinical literature usable in a systematic review to accumulate.

Quantitative synthesis was not possible. No two studies measured the same analytes at the same time points, sample sizes ranged from 1 to 300, and outcome definitions were investigator-defined throughout; effect estimates are therefore presented individually and never pooled. The certainty of the body of evidence was not formally rated, and risk of bias due to missing results was not assessed; both omissions are declared here and marked as such in the PRISMA checklist. This is a limitation of the review and is retained as such: the reasons for not applying a structured certainty framework are given in Section 2.7, but an acknowledgement is not a substitute for an assessment, and the reader is accordingly advised that the strength of the evidence underlying each finding reported here has not been graded. The methodological quality of the included evidence is limited: one study carries a critical risk of bias, four a serious risk, and no included study is randomised. Two studies declare industry funding or manufacturer-related conflicts of interest. Two of the three studies bearing most directly on the review’s central themes—the microbiological profile and the systemic inflammatory response—have design features that materially weaken them, namely a comparator group defined by its own outcome and differing five-fold in burn size [17], and a comparison between two enzymatically treated groups rather than against surgery [18].

## 6. Conclusions

Bromelain-based enzymatic debridement is associated with a measurable systemic response in burn patients: inflammatory markers rise more than after surgical debridement, early fever occurs in roughly one in seven patients and predicts subsequent wound infection, and, in the single study that analysed the treated area as a continuous exposure, the probability of a coagulation alteration increased with the area treated. None of these signals is presently explicable, because every mechanism proposed for them is a statement about tissue, and the tissue has not been examined: the human histopathological evidence amounts to nine patients and is entirely qualitative, while the selectivity that justifies the treatment has been demonstrated only in swine. Across 559 records and twelve included studies covering 979 patients, no study identified by the present search has evaluated the systemic and the tissue-level response in the same patients. Closing that gap requires a study design rather than a new technique: the same patients, quantitative histology at fixed time points, a systemic panel sampled on the same schedule, and observation extended beyond the first week.

## Figures and Tables

**Table 1:** Eligibility criteria, expressed as a PICOS framework. The population, intervention, comparator, study design, time frame and language criteria are common to both evidence streams; the outcome criterion is stream-specific.

Criterion	Included	Excluded
Population (P)—common to both streams	Human adult patients with thermal burns of any depth and extent, treated in a clinical setting.	Animal or ex vivo models; non-thermal wounds (chemical, electrical, necrotizing soft-tissue infection) when not separately reported.
Intervention (I)—common to both streams	Bromelain-based enzymatic debridement (NexoBrid^®^, or bromelain-based debriding agent), applied to the burn wound.	Other debriding agents (collagenase, papain-urea, maggot therapy); studies in which enzymatic debridement was not administered or its use cannot be attributed to the reported population.
Comparator (C)—common to both streams	Any comparator or none. Studies without a comparator group were eligible; where a comparator was present it was most often surgical debridement or standard of care.	No study was excluded for the absence of a comparator.
Outcomes (O)—Stream A (stream-specific)	At least one clinical outcome reported together with systemic biological parameters (inflammatory, coagulation, haematological or biochemical).	Clinical outcomes reported in isolation, without any systemic biological data.
Outcomes (O)—Stream B (stream-specific)	At least one clinical outcome reported together with histopathological findings obtained from human burn tissue.	Clinical outcomes reported in isolation, without any histopathological data.
Study design (S)—common to both streams	Original clinical investigations: randomised controlled trials, prospective cohorts, retrospective cohorts, case series and case reports with individual-level data.	In vitro and laboratory experimental studies; narrative reviews; systematic reviews (screened for reference harvesting only); editorials, letters without original data, conference abstracts; expert opinion.
Time frame—common to both streams	Published in the last ten years (2016—search date), reflecting the period of routine clinical availability of the agent in Europe.	Published before 2016.
Language—common to both streams	English.	Publications in other languages without an English full text.

**Table 2:** Characteristics of the twelve included studies, ordered chronologically by year of publication; Di Lonardo 2018 [19] and Miura 2025 [20] constitute Stream B and the remaining ten studies Stream A. All twelve full texts were obtained and read.

Study/Country	Design	*n*	Age	TBSA/Depth	Enzymatic Debridement Protocol	Comparator
Di Lonardo 2018 [19] Italy (Pisa)	Prospective case series with paired pre-/post-debridement biopsies	8	Mean 49 y; 5 M/3 F	Mean TBSA ≈30%; mid-deep and deep burns; aetiology not reported; biopsied wounds on the anterior lower limbs	NexoBrid^®^ within the first 24 h after injury; maximum 15% TBSA per application; gel pre-spread on a non-adhesive dressing to prevent sliding; contact time not stated	None
Martín 2018 [26] Spain (Barcelona)	Case report	1	41 y (M), 89 kg	15% TBSA; intermediate/deep second-degree, no full thickness; ABSI 7	6% TBSA (forearms), day 1 after admission, 4 h application, under deepened sedation (already intubated)	None
Hofmaenner 2021 [18] Switzerland (Zurich)	Retrospective single-centre study	59 (RG 49/ OG 10)	Median 42 y (31–59); 72.9% M	Overall TBSA 13% (6–26); RG 11.5% (5–16) vs. OG 42.75% (24–65), *p* < 0.0001. ABSI 5 vs. 8.5 (*p* = 0.0002); Baux 54 vs. 105.75 (*p* < 0.0001). Depth: superficial dermal 50.8%, deep dermal 49.2%, no ful thickness	NexoBrid^®^ in a single session, median 1 day after injury (1–3) with no difference between groups (*p* = 0.295); treated area RG 6% (2.5–9.5) vs. OG 18% (15–19) TBSA; general anaesthesia in 86.4%, regional block in 7 patients; all treated in the ICU	Internal comparison only: <15% vs. >15% TBSA treated in one session. No surgical control arm; the two groups differ markedly in burn severity
Sharaf 2022 [17] United Kingdom (Wakefield)	Retrospective comparative study	63 (40ED/23 non-ED)	ED mean 45.7 y (18–80) vs. 44 y (21–82)	ED mean TBSA 11.78% (0.25–50%) vs. 2.08% (0.1–8%); flame 77.5% vs. 21.7%; 13 ED patients with TBSA ≥ 15%; after ED, deep dermal or full-thickness areas in 82.5%	NexoBrid^®^ at a mean of 1.35 days post-injury (range <24 h to 3 days); no routine antibiotics, single preoperative prophylactic dose only	Adults managed by surgical debridement or dressings alone during the same period whose wounds took more than three weeks to heal (*n* = 23). Groups not comparable: the authors state that TBSA, mechanism and anatomical distribution differ substantially
Hasham 2023 [27] United Kingdom (Nottingham/ Liverpool)	Case series	2	43 y (F); 61 y (M)	Case 1: 31% mixed-depth flame; Case 2: 1.75% deep dermal flame	Case 1: 40 g at 24 h post-injury, 25% TBSA in one ICU session, prophylactic tranexamic acid 1 g t.d.s. to 48 h. Case 2: 2 g under popliteal sciatic nerve block; last rivaroxaban dose 32 h before	None
Pfister 2023 [14] Switzerland (Zurich)	Retrospective two-group cohort	132 (66ED/66 SD) 188 assessed	Median 46 y (34–60); ED 49.5 (33–59) vs. SD 45.0 (35–61), *p* = 0.716; 77% M	Median TBSA 17.0% (11.9–29.0), *p* = 0.785 between groups; ABSI 6 (5–8); Baux 65.8; inclusion TBSA ≥ 1%	NexoBrid^®^ per European consensus indication; timing, treated area and number of applications not reported	Regular surgical debridement, *n* = 66. Not matched; groups compared for baseline comparability
Capitelli-McMahon 2024 [28] United Kingdom (Wakefield)	Retrospective matched cohort	52 (26ED/26 SOC) 31 treated	Mean 44.5 y (ED) vs. 44 y (SOC)	Mean TBSA 35.5% (ED) vs. 35.8% (SOC); mean TBSA debrided in the analysed session 17% (range 2–45%) vs. 16% (3–55%); deep partial- and full-thickness	NexoBrid^®^ in the ICU; median 1 day from injury (vs. 4 days for SOC); always within the first four days, 14/26 on the day of injury or the next; single application in all but one patient	Standard-of-care surgical debridement in theatre, matched on age, %TBSA and inhalation injury (Baux components); inhalational match 21/26; 22/26 SOC patients had more than one theatre trip
Deplazes 2024 [16] Switzerland (Zurich)	Retrospective cohort	269 (83ED/186 no ED)	Median 50 y (IQR 33–65); 26% F	Median TBSA 19% (IQR 11–30); ABSI 6 (5–8); Baux 70 (52–93); inhalation injury 18%	NexoBrid^®^ within 2–3 days of injury (day 1 for circumferential burns); exposure consistently 4 h; treated area not reported	Contemporaneous ICU burn patients not treated with bromelain (*n* = 186); regression-adjusted for nine covariates, not matched
Breidung 2025 [15] Germany (Nuremberg)	Retrospective single-centre pre–post observational study	75	Mean 48.6 ± 18.9 y; 69.3% M	Mean TBSA 4.6 ± 2.9% (cohort restricted to <10%); third-degree in 18.7%; inhalation injury 10.7%	NexoBrid^®^ 4 h; ≤3% BSA in 57.3% and >3% BSA in 42.7%; local infiltration, plexus block, sedation or general anaesthesia according to site and size	None external; within-patient pre–post and ≤3% vs. >3% BSA subgroups
Bulla 2025 [11] Spain (Barcelona)	Retrospective cohort of the first 300 consecutive patients	300	Median 41 y (18–94); 76% M	Median TBSA burned 8% (0.5–98); median TBSA treated 5% (0.5–43). Pre-ED clinical depth: deep dermal 59.7%, intermediate dermal 27.7%, third-degree 12.3%. Flame 46.7%, deflagration 21.3%, scald 18.7%	NexoBrid^®^ within the first five days; 27.6% on the day of injury and 47.5% at 24–48 h; ≤15% TBSA per session with ≥24 h between sessions; 2 h polihexanide pre-soak, 1.5–3 mm layer, 4 h occlusion; regional block or general anaesthesia. Sequential ED in 26 patients (median 24.6% TBSA, maximum 43%), completed by day 5 in all	None. Within-patient comparison of post-ED grade against the admission clinical assessment; the authors declare the absence of a standard-of-care control group as a limitation
Kaita 2025 [29] Japan (Tokyo)	Retrospective cohort with propensity-score overlap weighting	17 (7ED + surgery/10 surgery)	Median 59 y (IQR 50–71); ED group 54 (25–60)	Median TBSA 46% (IQR 34–55); full-thickness area 22%; Burn Index 35; all flame; inhalation injury 65%	NexoBrid^®^ on the day of injury (6/7) or the next day (1/7); 30% TBSA per session (15% in one case); 4 h under polyurethane film then 2 h saline gauze; two applications in one case; ketamine analgesia in all	Conventional surgical excision alone (*n* = 10)
Miura 2025 [20] Japan (Gifu)	Case report	1	53 y, M; hypertension and dyslipidaemia	6% TBSA flame burn (welding sparks igniting clothing) of the right shoulder and upper arm; clinically assessed as deep dermal to full thickness; histologically classified as deep dermal	5 g NexoBrid^®^ in 50 g gel applied to 2.5% TBSA around the shoulder; overnight saline pre-soak; ED at 22 h post-injury; 4 h application; 2 h post-soak with 0.025% benzalkonium chloride; Mepilex Ag dressing; propofol and ketamine intravenously	None

ABSI, abbreviated burn severity index; ED, enzymatic debridement; F, female; IQR, interquartile range; M, male; OG, off-label group; RG, regular-use group; SD, surgical debridement; SOC, standard of care; and TBSA, total body surface area. The Baux score is the sum of age and percentage TBSA.

**Table 3:** Coagulation and haemostasis findings.

Study	Results	Bleeding/Transfusion	Authors’ Conclusion
Martín 2018 [26]	Pre-application: Hb 15 g/dL, platelets 165 × 10^9^/L, PT 76%, PT ratio 1.19, aPTT ratio 1.01, fibrinogen 3.58 g/L. At 24 h: Hb 9.8, platelets 110, PT 43%, PT ratio 1.78, aPTT ratio 1.24, fibrinogen 3.98. After 10 mg intravenous phytomenadione: PT 47%, ratio 1.74, aPTT 1.10, fibrinogen 4.64. At 48 h: PT 80%, ratio 1.16, aPTT 0.94, fibrinogen 5.85. At 72 h: PT 87%, ratio 1.10, aPTT 0.81, fibrinogen 5.86. Complete reversal by 48 h. The authors state the competing explanation themselves: cumulative 9840 mL crystalloid plus 800 mL 20% albumin, positive balance 17,239 mL	No active bleeding; no transfusion; a single 10 mg intravenous dose of phytomenadione	Coagulation-related adverse effects should be monitored after NexoBrid^®^; caution is advised in patients on antiplatelet or anticoagulant therapy
Hofmaenner 2021 [18]	Coagulation parameters were not among the reported outcomes. Bleeding was defined prospectively as a haemoglobin fall > 1 g/dL with clinical signs of blood loss; no bleeding event occurred in either group, and there was no difference in transfusion during the procedure	No bleeding events; no difference in blood transfusion between the regular-use and off-label groups	Enzymatic debridement produced no expected or unexpected side effects, including in patients treated over more than 15% TBSA in a single session
Hasham 2023 [27]	Case 1 (PT/aPTT/TT, s; Hb g/L): Admission 14.0/22.6/28.9, Hb 116; day 1 pre-ED 20.4/34.8/22.2, Hb 127; day 1 post-ED 20.7/35.7/22.3, Hb 119; day 8 11.9/27.7/22.1; day 55 13.3/25.5/26.3; day 65 13.1/25.0/26.0. Essentially unchanged across the application. Case 2: Admission clotting normal, values not given	Case 1: No bleeding complication, no blood products. Case 2: Two major haemorrhages from a single breached venous varicosity within the treated area—blood loss up to 2 litres, BP 50/40 mmHg, 2 units packed red cells and 1 g tranexamic acid, then cardiac arrest requiring two cycles of CPR and adrenaline; the vessel was ligated. The patient sustained a posterior cerebral artery infarct with left hemiparesis	Haemorrhage can occur; eschar overlying a large vessel or varicosity should be regarded as a relative contraindication. Enzymatic debridement can be used in patients with pre-existing clotting disorders without blood products, but haematological support is essential
Pfister 2023 [14]	Factor V was significantly higher in the enzymatic group across the seven days (*p* < 0.01): day 1, 97% (84–106) vs. 81% (66–91); day 4, 120% (107–154) vs. 86.5% (74–105); day 7, 142% (127–198) vs. 125.75% (113–132). No difference in INR (*p* = 0.21), aPTT (*p* = 0.37), fibrinogen (*p* = 0.54), factor XIII (*p* = 0.94) or platelets (*p* = 0.95). Haemoglobin reached its nadir on day 7 in both groups (89 g/L enzymatic vs. 90 g/L surgical), with no between-group difference (*p* = 0.06). Acute burn-induced coagulopathy (INR > 1.2 at any point in seven days) occurred in 63/132 patients—31 enzymatic, 32 surgical; within this subgroup the only difference was a higher proportion of IIa° burn area in the enzymatic patients (7.0% vs. 2.0%, *p* = 0.016)	No difference in the amount of blood-product transfusion between groups; both received similar fluid volumes	Debridement does not appear to increase the risk of coagulation abnormalities compared with the regular surgical approach. The authors acknowledge that an effect confined to larger burns could have been missed and that no power calculation was performed
Capitelli-McMahon 2024 [28]	Prothrombin time never breached the local reference range in either group; no significant difference (*p* = 0.0601). Both groups exceeded the upper aPTT limit on day 2: the enzymatic group by a mean of 1.9 s (mean 33.9 s against an upper limit of 32 s), with 10/26 patients above the limit, and the surgical group by a mean of 2.3 s, with 9/26 above; no significant difference (*p* = 0.2113). The enzymatic group fell below the lower platelet limit of 135 × 10^9^/L on day 2, to a mean of 132 × 10^9^/L; the surgical group did not; no significant difference (*p* = 0.5287)	No platelets, packed red cells or fresh frozen plasma were required by any of the 26 enzymatically debrided patients	No convincing evidence that enzymatic debridement is associated with coagulation abnormalities
Breidung 2025 [15]	Prothrombin ratio (Quick, % of normal) fell from 82.0 ± 13.3 to 76.8 ± 14.9 (*p* = 0.002). Haemoglobin fell from 13.3 ± 1.6 to 12.6 ± 1.7 g/dL (*p* < 0.001). aPTT showed no significant change	No bleeding requiring intervention in the cohort	Measurable systemic changes occur after NexoBrid^®^ even in burns under 10% TBSA, particularly in inflammatory and coagulation parameters; the changes were judged mild and not clinically significant
Bulla 2025 [11]	Coagulation testing before debridement in all 300 patients; post-treatment testing was not performed in 74. Among the 226 tested, 40% showed a coagulation alteration after debridement, most commonly a prolongation of prothrombin time. The probability of a coagulation alteration rose with the area treated: logistic regression on TBSA treated with enzymatic debridement gave an odds ratio of 1.158 per percentage point (95% CI 1.103–1.224; *p* = 2.83 × 10^−8^), and TBSA differed highly significantly between patients with and without alteration (*p* = 6.7 × 10^−12^)	22/300 patients (7%) required transfusion, median 2 units (range 1–6). Transfusion likewise scaled with treated area: odds ratio 1.13 per percentage point (95% CI 1.08–1.18; *p* = 5.4 × 10^−8^)	The relationship between treated area and coagulation alteration is confirmed, but the design cannot distinguish a direct effect of bromelain on the coagulation cascade from consumption of clotting factors through blood loss. The authors interpret the pattern as depletion of vitamin K-dependent factors and have since added vitamin K supplementation to their protocol for areas > 10% TBSA

aPTT, activated partial thromboplastin time; BP, blood pressure; CPR, cardiopulmonary resuscitation; ED, enzymatic debridement; Hb, haemoglobin; INR, international normalised ratio; PT, prothrombin time; TBSA, total body surface area; and TT, thrombin time.

**Table 4:** Infection, bacteraemia and wound microbiological profile.

Study	Method	Results	Effect Estimate and Conclusion
Hofmaenner 2021 [18]	Wound infection recorded as a clinical outcome	Wound infection in 6/49 (12.2%) of the regular-use group and 4/10 (40%) of the off-label group (*p* = 0.06)	No formal effect estimate; the difference is attributed to burn severity rather than to the procedure
Sharaf 2022 [17]	Surface wound swabs on admission, at debridement and weekly, compared against a non-enzymatically treated cohort	Before debridement, 55% of enzymatically treated patients had no bacterial growth; in the first week after debridement, 47.5% had none. Gram-positive organisms predominated in week 1 (S. aureus in 27.5%) and Gram-negative in week 2 (30% of patients, *P. aeruginosa* in 22.5%), returning to Gram-positive predominance in week 3. Of the 12 patients with Gram-negative emergence in week 2, seven had TBSA ≥ 15%. Clinical local and systemic infection developed in 2/40 patients, requiring intravenous antibiotics, with one requiring surgical washout. In the comparator cohort, *S. aureus* emerged in 73.9%, coliforms in 34.8% and *P. aeruginosa* in 8.7%, and 3/23 developed localised infection controlled with oral antibiotics	No effect estimate is reported: the study contains no *p*-value, confidence interval or adjusted comparison. There is no strong evidence that NexoBrid^®^ changes the profile of bacterial colonisation, and the study states explicitly that the two cohorts differ in TBSA (mean 11.78% vs. 2.08%), mechanism and anatomical distribution
Deplazes 2024 [16]	Blood cultures on clinical indication; competing-risk (Fine–Grey) analysis with death as the competing event	First bacteremia in 61/269 (23%), yielding 85 pathogens; median time to first bacteremia 6 days (IQR 5–11); polymicrobial in 14/61 (23%); 51 (60%) Gram-positive and 34 (40%) Gram-negative. Bacteremia 22/83 (27%) with bromelain vs. 39/186 (21%) without. Commonest isolates: *S. aureus* 16 (19%), *K. pneumoniae* 7 (8%), *S. mitis/oralis* 7 (8%), *P. aeruginosa* 6 (7%). Multidrug resistance in 5 pathogens, 3 of them in bromelain-treated patients	Fully adjusted subdistribution hazard ratio 0.79 (95% CI 0.42–1.48; *p* = 0.47). ICU mortality HR 0.55 (95% CI 0.26–1.20; *p* = 0.14). Enzymatic debridement is unlikely to be associated with an increased risk of bacteremia
Bulla 2025 [11]	Early post-ED fever and clinically diagnosed infection recorded prospectively in the medical record; pathogens identified on culture	Early post-debridement fever in 14% of patients. Fever was more frequent in younger patients (Wilcoxon *p* = 0.0483; logistic regression odds ratio 0.977 per year of age, 95% CI 0.954–0.998), and showed no association with sex, day of debridement, clinical burn depth or post-ED grade. Infection occurred in 42/300 patients; the commonest pathogens were *S. aureus* (19 isolates), *P. aeruginosa* (17), *E. faecalis* (4), *E. coli* (3), *E. cloacae* (3), *K. pneumoniae* (3) and *C. albicans* (2)	Early fever predicted later wound infection: Fisher’s exact *p* = 0.0256, odds ratio 2.5 (95% CI 1.09–5.49). The authors attribute post-ED fever primarily to an inflammatory response rather than to bacteraemia, but recommend blood cultures and S. aureus screening in every febrile patient
Kaita 2025 [29]	Blood cultures; propensity-score overlap weighting for age, sex, inhalation injury and TBSA	Bacteraemia in 5/7 (71%) with combined enzymatic and surgical debridement vs. 9/10 (90%) with surgical excision alone. Organisms: *Pseudomonas aeruginosa*, *Stenotrophomonas maltophilia*, *Candida* spp., *Enterococcus* spp. Transfusion volumes did not differ: red cells 3080 mL (IQR 1680–4480) vs. 3360 mL (280–3920), *p* = 0.84	Weighted odds ratio for bacteraemia 0.06 (95% CI 0.00–0.96; *p* < 0.05). Combining surgical excision with enzymatic debridement may reduce the time to complete debridement and the rate of bacteraemia

**Table 5:** Inflammatory, biochemical and haematological findings.

Study	Parameters and Results	Time Course	Authors’ Interpretation
Hofmaenner 2021 [18]	CRP means 135.7 mg/L (regular group) vs. 228.7 (off-label), difference −92.94 (95% CI −132.8 to −53.11), overall *p* = 0.5362. The only significant difference occurred at baseline, 24 h before debridement (adjusted *p* = 0.0016), with lower levels in the group with the smaller burn; after debridement no difference was detectable on any of the three days (*p* = 0.2913, 0.3701, 0.0848). Leukocytes *p* = 0.6306, procalcitonin *p* = 0.6327, lactate *p* = 0.5505, central venous oxygen saturation *p* = 0.20. Electrolytes showed no shift, with a single isolated exception (maximum potassium on day 1, adjusted *p* = 0.0081). Heart rate differed on days 1–3 (*p* = 0.0048, 0.0283, 0.0157) and intraoperative fluid volume was higher in the off-label group (475 vs. 700 mL, *p* = 0.0393)	−24 h, +24 h, +24–48 h, +48–72 h	No systemic inflammatory response was detectable—a result the authors describe as contradicting earlier reports of post-bromelain fever, and speculatively attribute to anti-inflammatory properties of bromelain. Higher heart rates and fluid requirements are ascribed to the larger wound area and the hypermetabolic state
Pfister 2023 [14]	CRP and procalcitonin were significantly higher in the enzymatically debrided group (both *p* < 0.01). Median CRP (mg/L), surgical vs. enzymatic: day 3, 130 vs. 151; day 4, 155 vs. 208; day 5, 137 vs. 213; day 6, 129 vs. 220; day 7, 117 vs. 183. Median procalcitonin (ng/mL): day 1, 0.06 vs. 0.08; day 2, 0.19 vs. 0.18; day 3, 0.36 vs. 0.98. No difference in leukocytes (*p* = 0.59), lactate (*p* = 0.50), pH (*p* = 0.11), bilirubin (*p* = 0.22), AST (*p* = 0.37), ALT (*p* = 0.47) or GGT (*p* = 0.29). Body temperature and fluid volumes did not differ (*p* = 0.35 and *p* = 0.92)	Daily, days 1–7	The higher acute-phase protein levels are attributed to an increased local inflammatory tissue reaction producing a greater systemic response. This heightened inflammation could underlie the observed factor V elevation, and the correlation remains to be investigated
Breidung 2025 [15]	CRP rose from 4.8 ± 5.8 to 10.8 ± 7.2 mg/dL (*p* < 0.001). Haemoglobin fell from 13.3 ± 1.6 to 12.6 ± 1.7 g/dL (*p* < 0.001). Prothrombin ratio fell from 82.0 ± 13.3 to 76.8 ± 14.9 (*p* = 0.002). Body temperature rose from 36.8 ± 0.5 to 37.0 ± 0.7 °C (*p* = 0.018). Leukocyte count unchanged, 11.8 ± 5.0 to 11.8 ± 5.9 (*p* = 0.927). Pain unchanged, NRS 2.3 ± 1.9 to 2.7 ± 2.2 (*p* = 0.340). aPTT, sodium, potassium and creatinine unchanged. Subgroup: body temperature slightly higher with >3% BSA treated (*p* = 0.036)	Two time points only, within 24 h before and within 24 h after application	Measurable systemic changes occur even in burns under 10% TBSA, particularly in inflammatory and coagulation parameters; the changes are mild and support safe use
Bulla 2025 [11]	Early post-debridement fever in 14% of patients, inversely related to age; no relation to burn depth or to the extent of debridement	Throughout admission	Post-ED fever is attributed primarily to an inflammatory response, supported by its predominance in younger patients, in whom a more robust inflammatory response is expected
Kaita 2025 [29]	No blood or laboratory analyte was measured or reported anywhere in the study. The only physiological measurements are intratissue (compartment) pressures in two cases: case 5, left hand 52 → 20 mmHg and right hand 36 → 15 mmHg; case 6, left upper arm 81 → 12 mmHg and left forearm 55 → 5 mmHg	Before and 4 h after application	Not interpreted as a biological endpoint by the authors; reported as evidence of decompression of the burned compartment

**Table 6:** Clinical outcomes.

Study	Debridement	Grafting and Wound Closure	Length of Stay	Mortality	Scar Outcome
Martín 2018 [26]	Single 4 h application to 6% TBSA	Not reported	Extubated day 4; discharged day 16	Survived	Not reported
Hofmaenner 2021 [18]	Single session; median 1 day after injury; time to surgical wound cover 30 min (regular) vs. 65 min (off-label), *p* = 0.024	Skin grafting in 30/49 (61.2%) of the regular-use group vs. 5/10 (50%) of the off-label group (*p* = 0.510)	Hospital 19 days (10–32); regular 16 vs. off-label 40.5	Survival 49/49 (100%) regular vs. 7/10 (70%) off-label, *p* = 0.004	Not reported
Sharaf 2022 [17]	Debridement at a mean of 1.35 days; after debridement, deep dermal or full-thickness areas remained in 82.5%	Autografting required in 65% (26/40); wounds not progressing at two weeks were grafted	Time from first to last swab, i.e., to healing: mean 6 weeks (range 2–28) vs. 5.7 weeks (3–18) in the comparator	2/40 died, in weeks 5 and 7	Not reported
Hasham 2023 [27]	Single application in each case	Case 1: split-skin grafting of 8% TBSA, uncomplicated. Case 2: not reported	Not reported (case 1 followed to day 65)	No deaths reported	Not reported
Pfister 2023 [14]	Not reported as an outcome	Not reported	ICU 7 days (3–18); hospital 20.5 days (12–34); no difference between groups (*p* = 0.916 and *p* = 0.265)	13/132 (10%): 5 enzymatic, 8 surgical (*p* = 0.559)	Not reported
Capitelli-McMahon 2024 [28]	Single application in all but one patient; median 1 day from injury vs. 4 days for surgical controls; 22/26 surgical controls needed more than one theatre trip	Not reported	Not reported	Not reported	Not reported
Deplazes 2024 [16]	Median 2 operations (IQR 2–3, range 0–19); median time to first surgery 2 days (IQR 1–4)	Not reported	Not reported	ICU mortality 40/269 (15%); 9/83 (11%) with bromelain vs. 31/186 (17%) without	Not reported
Breidung 2025 [15]	Single 4 h application	Not reported	11.5 ± 13.0 days	3/75 (4.0%): two from pneumonia-associated septic multiorgan failure after inhalation trauma, one with pre-existing cirrhosis	Not reported
Bulla 2025 [11]	Post-ED grading reclassified depth in a large proportion of patients; agreement with the admission clinical assessment was only slight to fair (quadratic weighted Cohen’s κ = 0.291, *p* < 0.001), the initial evaluation usually overestimating depth. Emergency escharotomy was needed in 7/300	Surgery by post-ED grade: 32.7% of 2A, 54.1% of 2B, 81.4% of 2BE, 95.1% of grade 3. Logistic regression against grade 2A: 2B odds ratio 2.43 (1.25–4.85), 2BE 8.98 (3.85–22.39), grade 3 40.15 (10.61–265.37); McFadden R^2^ = 0.151, against 0.052 for the admission clinical assessment. Median healing time 37 days (15–281); 33 days without surgery vs. 40 with (*p* = 0.00699)	Not reported	Not reported	Hypertrophy in 58/300 (99 records incomplete). Surgery raised the odds of hypertrophy 3.64-fold (1.69–8.44, *p* = 0.0004); post-ED grade 2BE raised them 3.67-fold (1.44–10.11, *p* = 0.008) and grade 3 3.08-fold (1.00–9.84, *p* = 0.052), whereas the admission clinical grade showed no association. Contracture surgery in 31/267; primary grafting raised the odds 3.53-fold (1.36–10.86, *p* = 0.00438)
Kaita 2025 [29]	Time to complete debridement 2 post-burn days (IQR 2–4) vs. 6 (IQR 4–7), *p* = 0.009; weighted difference −4 days (95% CI −5 to −2)	MEEK micrografting plus autologous cell suspension, grafting 2 days after removal; closure at 2–3 months in the three cases described individually	ICU stay 77 days (IQR 65–119) vs. 69 (35–114), *p* = 0.46	In-hospital 2/7 (29%) vs. 3/10 (30%); weighted odds ratio 0.93 (0.10–8.35, *p* = 0.95)	Not assessed; the authors state that functional and cosmetic evaluation has not yet been performed

**Table 7:** Histopathological findings in the two human studies.

Item	Di Lonardo 2018 [19] (*n* = 8)	Miura 2025 [20] (*n* = 1)
Biopsy protocol	18 specimens: 8 before and 10 after debridement. Pre-debridement specimens taken immediately before gel application; post-debridement specimens shortly after debridement; in one patient two further specimens at 24 h. Technique: 4 mm punch, plus two elliptical skin lozenges approximately 3 cm long deliberately spanning macroscopically different zones (off-white and ecchymotic). Site: Anterior surface of the lower limbs	One specimen only, taken on day 10 post-injury—nine days after debridement and four days after pseudoeschar was first seen clinically on day 6—immediately before surgical debridement. No pre-debridement biopsy. Technique: Incisional biopsy, a spindle-shaped specimen including subcutaneous adipose tissue, taken from the wound margin of the right upper arm so as to encompass both healthy and burned skin in a single section
Processing and stains	Immediate fixation in 10% neutral formalin; haematoxylin–eosin only. No immunohistochemistry	Four stains: Haematoxylin–eosin; Elastica–Masson (collagen bright green, elastic fibres dark violet); Alcian blue; and periodic acid–Schiff. No immunohistochemistry. Fixation protocol not stated
Assessment method	Purely qualitative descriptive histopathology. No scoring system, no grading scale, no morphometry, no image analysis, no blinding, no inter-observer assessment. Depth of necrosis described but never measured	Purely qualitative descriptive histopathology. No scoring system, no grading scale, no morphometry. Burn depth was nevertheless classified histologically, as a deep dermal burn, on the basis of the vascular and adnexal findings
Pre-debridement findings	Completely de-epithelialised areas. Foci of coagulative necrosis unevenly distributed throughout the dermis. Skin adnexa either partly preserved or showing cytological alterations attributable to thermal damage (pyknotic nuclei with a perinuclear halo). Inflammatory cells almost constantly present, predominantly neutrophils, with interstitial or perivascular distribution	None. No baseline histology exists for this patient
Post-debridement findings	Homogenization of the upper dermal remnant was the most significant feature, whereas the deep dermis retained its morphological features and appeared normal. Residual thermal necrosis persisted in the most superficial dermis alongside partially viable adnexa. In macroscopically ecchymotic areas, marked vascular congestion with frequent blood extravasation from capillary disruption. The few remaining adnexal structures were poorly viable. Numerous inflammatory cells, more abundant in the deep dermis. The authors interpret the homogenised layer as a scaffold progressively revitalised from below	The lower reticular dermis appeared preserved. Capillaries in the superficial reticular dermis were absent, while those in the deeper layers remained intact, with inflammatory cell infiltration around the deeper capillaries—the finding on which the histological classification as a deep dermal burn was based. An ulcer layer composed mainly of inflammatory cells occupied the upper dermis, with a further layer above it containing few inflammatory cells: the pseudoeschar
Pseudoeschar	The term is not used. The analogous entity is described clinically as a “neo-eschar” formed when the residual homogenised dermis desiccates, which either detaches slowly and permits re-epithelialization or deepens and requires surgical removal. No histological criteria are given, since the biopsies were taken at debridement rather than at the neo-eschar stage	The core contribution of the study. On Elastica–Masson the pseudoeschar consisted principally of collagen fibres with elastic fibres also present; Alcian blue and PAS were negative, excluding mucopolysaccharides and glycans. Inflammatory cells were seen spiralling upward from the lower dermis into the uppermost layer, penetrating the ulcer layer, and collagen and elastic fibres ascended along the same route. Distinguished from residual eschar by composition and directionality: newly extruded degenerated dermal matrix migrating upward rather than retained thermally coagulated tissue lying passively on the surface. Proposed mechanism: Transepithelial elimination of degenerated dermal components presumed to originate in the zone of stasis, analogous to acquired reactive perforating collagenosis
Quantitative measurements	None. No thickness in µm or mm, no percentages, no cell counts, no statistics	None. No thickness, no percentages, no cell counts, no statistics
Correlation with clinical outcome	Narrative and mechanistic only. The authors link the histological picture to the clinical need to prevent desiccation of the residual dermis, and recommend early homografting with later autograft completion over full-thickness areas. No per-patient outcome data, no healing times, no graft rates, no scar scores, no statistical correlation	Narrative. Surgical debridement with Versajet on day 10 exposed subcutaneous fat in part; split-thickness grafting (3:1 mesh) with autologous cell suspension followed; epithelialisation was largely complete by day 25 and the patient was discharged on day 30. The authors argue retrospectively from the histology that, because the lower dermis was viable and no fat or vessels were exposed after debridement, spontaneous re-epithelialisation might have occurred had the pseudoeschar been allowed to detach—that is, the histology suggests the operation may not have been necessary. Once the pseudoeschar had formed, the patient reported complete pain relief and needed no sedation for dressing changes, which the authors read as a protective function
Systemic or laboratory data reported alongside histology	None. No blood counts, no inflammatory markers, no biochemistry, no coagulation studies, no microbiology	None. The only systemic statement in the entire report is the observation that there were no signs of systemic infection. No blood count, inflammatory marker, biochemical or coagulation value is given at any point
Self-declared limitations	None. The paper contains no limitations section and does not acknowledge the small sample, the absence of a control, the qualitative assessment, the restricted anatomical sampling or the lack of follow-up	Extensive and explicit: A single case; histological evaluation at only one time point; the natural course without surgery is unknown; the findings reflect tissue conditions ten days after application; and—importantly—1% silver sulfadiazine had been applied daily from day 6, so its known capacity to induce a pseudoeschar of its own cannot be separated from the effect of enzymatic debridement, and it may have altered the surface morphology or composition of the layer examined

**Table 8:** Risk of bias in the included studies, assessed with ROBINS-I [24] for comparative and cohort designs and with the JBI critical appraisal checklists [25] for case series and case reports.

Study	Instrument	Dominant Concerns	Overall Judgement	Funding/Conflicts
Di Lonardo 2018 [19]	JBI case series	Purely qualitative assessment with no scoring instrument, no blinding and no inter-observer assessment; restricted anatomical sampling; no follow-up; a single stain. The paper contains no limitations section and declares no funding or conflicts	Moderate—JBI 6/8 (75%)	Not stated
Martín 2018 [26]	JBI case report	Single patient. The authors themselves identify a competing explanation for the coagulation derangement—a cumulative positive fluid balance of 17,239 mL—which cannot be separated from a drug effect	High risk by design; transparent reporting	None declared
Hofmaenner 2021 [18]	ROBINS-I	The exposure under comparison is the treated area, which is itself a proxy for burn severity: the off-label group had four times the TBSA (42.75% vs. 11.5%), higher ABSI (8.5 vs. 5) and higher Baux score (105.75 vs. 54). Severity therefore confounds every outcome, including the reported survival difference. There is no surgical control arm. Two of the four significant findings (baseline CRP, intraoperative fluid volume) reflect this imbalance rather than the intervention	Serious	No funding declared. One author is a consultant for MediWound Germany and three others have received travel support from the manufacturer
Sharaf 2022 [17]	ROBINS-I	The comparator was defined by its outcome—adults whose wounds took more than three weeks to heal—and differs from the enzymatic group five-fold in mean TBSA (11.78% vs. 2.08%), in mechanism (flame 77.5% vs. 21.7%) and in anatomical distribution. No statistical test, *p*-value or confidence interval appears anywhere in the paper, and no adjustment was attempted	Critical	No funding; no conflicts declared
Hasham 2023 [27]	JBI case series	Two cases selected precisely because they were informative about bleeding; by construction not representative. Laboratory reporting for case 1 is complete and transparent	High risk by design; adequate reporting	No funding; no conflicts declared
Pfister 2023 [14]	ROBINS-I	Allocation to enzymatic or surgical debridement was made by the treating surgeon on the basis of burn depth distribution, so confounding by indication is not formally addressed; no matching or multivariable adjustment was applied, although the groups proved comparable on age, BMI, ABSI, Baux score and TBSA. Fifty-six of 188 patients were excluded. No power calculation was performed, and the authors acknowledge that an effect confined to larger burns could have been missed. Outcome measurement is objective and the analytical panel was prespecified	Moderate	No commercial or financial relationships declared
Capitelli-McMahon 2024 [28]	ROBINS-I	Two concerns dominate. The control group was drawn from 2011 to 2020 while the enzymatic group came from 2017 to 2020, so the comparison spans different eras of burn care. Twenty-two of 26 control patients underwent several debridement procedures, and the single session analysed for each was chosen post hoc as the one most closely matching its enzymatic counterpart in extent and timing—selection of the analysed exposure by the investigators. Five of 31 eligible patients were excluded	Serious	None declared
Deplazes 2024 [16]	ROBINS-I	Large cohort with adjustment for nine covariates and an appropriate competing-risk model. Residual confounding by indication remains. Blood cultures were drawn on clinical indication rather than by protocol, which may differ between groups	Moderate	Swiss National Science Foundation and university/foundation grants; funders had no role; no conflicts declared
Breidung 2025 [15]	JBI case series/ROBINS-I (self-controlled)	Pre–post design without a control group: the CRP rise, haemoglobin fall and prothrombin-ratio fall over 24 h cannot be separated from the natural evolution of the burn, from fluid resuscitation, or from concurrent care	Serious for attribution; low for measurement	Supported in part by PolyMedics Innovations GmbH, stated to have had no role in design, analysis or reporting; no conflicts declared
Bulla 2025 [11]	JBI case series/ROBINS-I (uncontrolled)	Large consecutive series with ethics approval and a minimum two-year follow-up, but no standard-of-care control group, which the authors declare. Missing data are substantial for two of the endpoints of interest to this review: no post-debridement coagulation test was performed in 74 of 300 patients (24.7%), and the hypertrophy field was incomplete in 99 of 300 (33%). Fever and aetiology findings were secondary to the primary aim, and the authors describe them as observations meriting dedicated study	Moderate; serious for the coagulation endpoint because of missing data	No funding; no conflicts declared
Kaita 2025 [29]	ROBINS-I	Seventeen patients, seven exposed. Propensity-score overlap weighting on four covariates in a sample of this size cannot achieve balance and produces unstable estimates; the bacteraemia odds ratio of 0.06 with an upper confidence bound of 0.96 is significant but extremely imprecise. Groups differ substantially in age and inhalation injury at baseline	Serious	No external funding; no conflicts declared
Miura 2025 [20]	JBI case report	Single patient, single post-debridement time point, no baseline biopsy. The dominant threat to the central finding is declared by the authors themselves: 1% silver sulfadiazine was applied daily from day 6, and silver sulfadiazine is independently known to induce a pseudoeschar, so its contribution to the layer examined on day 10 cannot be excluded. Reporting is otherwise complete, with informed consent and clinical follow-up to day 30	Moderate—JBI 5/8 (62.5%); reporting quality higher than that score suggests	No financial support; no conflicts declared

**Table 9:** Outcome domains addressed by each included study. Capitelli-McMahon [28] reports only procedural outcomes and no healing, grafting or survival outcome, and Di Lonardo [19] reports clinical management recommendations without per-patient outcome data; both are scored “Partly”. The final column is the primary endpoint of this review.

Study	Clinical Outcomes	Systemic Biological	Histopathological	Both Biological and Histological
Di Lonardo 2018 [19]	Partly	No	Yes	No
Martín 2018 [26]	Yes	Yes	No	No
Hofmaenner 2021 [18]	Yes	Yes	No	No
Sharaf 2022 [17]	Yes	Yes	No	No
Hasham 2023 [27]	Yes	Yes	No	No
Pfister 2023 [14]	Yes	Yes	No	No
Capitelli-McMahon 2024 [28]	Partly	Yes	No	No
Deplazes 2024 [16]	Yes	Yes	No	No
Breidung 2025 [15]	Yes	Yes	No	No
Bulla 2025 [11]	Yes	Yes	No	No
Kaita 2025 [29]	Yes	Yes	No	No
Miura 2025 [20]	Yes	No	Yes	No
Total	10/12	10/12	2/12	0/12

## Data Availability

No new data were created in this study. All data analysed in this review are contained in the published articles cited. The data extraction forms and the completed PRISMA 2020 and SWiM checklists are provided as Supplementary Material.

## Supplementary Materials

The following supporting information can be downloaded at: https://www.mdpi.com/article/10.3390/medsci14060667/s1. Table S1: Complete search strategies; Table S2: Reports excluded at the full-text stage, with reasons; Table S3: Biological parameters measured and sampling schedule (Stream A); Table S4: Residual data gaps in the included studies after full-text retrieval; Table S5: PRISMA 2020 checklist; Table S6: SWiM reporting checklist. References [11,14,15,16,17,18,19,20,26,27,28,29,34,40,41,42] are cited in the Supplementary Materials.

## Abbreviations

The following abbreviations are used in this manuscript:

aPTT	Activated Partial Thromboplastin Time
CI	Confidence Interval
CRP	C-Reactive Protein
F	Female
ED	Enzymatic Debridement
ICU	Intensive Care Unit
INR	International Normalised Ratio
JBI	Joanna Briggs Institute
M	Male
OR	Odds Ratio
PCT	Procalcitonin
PT	Prothrombin Time
PRISMA	Preferred Reporting Items for Systematic Reviews and Meta-Analyses
ROBINS-I	Risk Of Bias In Non-randomised Studies of Interventions
SOC	Standard Of Care
SWiM	Synthesis Without Meta-analysis
TBSA	Total Body Surface Area
