# Supplementary material for: Systemic and Histopathological Effects of Bromelain-Based Enzymatic Debridement in Human Burns—A Systematic Review
Source: Med Sci (Basel). 2026 Oct 13;14(6):667. doi: 10.3390/medsci14060667 (PMC13707106; doi:10.3390/medsci14060667)
Supplement: Supplementary file 1 [file medsci-14-00667-s001.zip › medsci-4582380-supplementary.pdf]

SUPPLEMENTARY MATERIAL

Systemic and Histopathological Effects of Bromelain-based Enzymatic Debridement in Human Burns – A Systematic Review

Contents

Table S1 — Complete search strategies, by database and evidence stream
Table S2 — Reports excluded at the full-text stage, with reasons
Table S3 — Biological parameters measured and sampling schedule (Stream A)
Table S4 — Residual data gaps in the included studies after full-text retrieval
Table S5 — PRISMA 2020 checklist
Table S6 — SWiM reporting checklist

Table S1. Complete search strategies

S1.1 Stream A (biological) — PubMed/MEDLINE — 80 records retrieved, 6 retained

```
("NexoBrid"[Title/Abstract] OR Nexobrid[Title/Abstract]
 OR "bromelain-based enzymatic debridement"[Title/Abstract]
 OR "enzymatic debridement"[Title/Abstract]
 OR "enzymatic necrosectomy"[Title/Abstract])
AND
(burn*[Title/Abstract] OR "thermal injury"[Title/Abstract]
 OR "burn wound"[Title/Abstract])
AND
(clinical[Title/Abstract] OR outcome*[Title/Abstract] OR healing[Title/Abstract]
 OR graft*[Title/Abstract] OR epithelialization[Title/Abstract]
 OR infection[Title/Abstract] OR bacteremia[Title/Abstract]
 OR bleeding[Title/Abstract] OR "blood loss"[Title/Abstract]
 OR biomarker*[Title/Abstract] OR laboratory[Title/Abstract]
 OR inflammatory[Title/Abstract] OR inflammation[Title/Abstract]
 OR coagulation[Title/Abstract] OR CRP[Title/Abstract]
 OR "C-reactive protein"[Title/Abstract] OR procalcitonin[Title/Abstract]
 OR leukocyte*[Title/Abstract] OR platelet*[Title/Abstract]
 OR fibrinogen[Title/Abstract] OR INR[Title/Abstract]
 OR aPTT[Title/Abstract] OR D-dimer*[Title/Abstract]
 OR albumin[Title/Abstract] OR hemoglobin[Title/Abstract])
```

S1.2 Stream A (biological) — Embase — 103 records retrieved, 10 retained

```
('nexobrid'/exp OR nexobrid:ti,ab
 OR 'bromelain based enzymatic debridement':ti,ab
 OR 'enzymatic debridement':ti,ab
 OR 'enzymatic necrosectomy':ti,ab)
AND
('burn'/exp OR burn*:ti,ab OR 'thermal injury':ti,ab OR 'burn wound':ti,ab)
AND
(clinical:ti,ab OR outcome*:ti,ab OR healing:ti,ab OR graft*:ti,ab
 OR epithelialization:ti,ab OR infection:ti,ab OR bacteremia:ti,ab
 OR bleeding:ti,ab OR 'blood loss':ti,ab OR biomarker*:ti,ab
 OR laboratory:ti,ab OR inflammatory:ti,ab OR inflammation:ti,ab
 OR coagulation:ti,ab OR crp:ti,ab OR procalcitonin:ti,ab
 OR leukocyte*:ti,ab OR platelet*:ti,ab OR fibrinogen:ti,ab
 OR inr:ti,ab OR aptt:ti,ab OR d-dimer*:ti,ab
 OR albumin:ti,ab OR hemoglobin:ti,ab)
```

S1.3 Stream A (biological) — Scopus — 76 records retrieved, 8 retained

```
TITLE-ABS(Nexobrid OR "bromelain-based enzymatic debridement"
 OR "enzymatic debridement" OR "enzymatic necrosectomy")
AND
TITLE-ABS(burn* OR "thermal injury" OR "burn wound")
AND
TITLE-ABS(clinical OR outcome* OR healing OR graft* OR epithelialization
 OR infection OR bacteremia OR bleeding OR "blood loss" OR biomarker*
 OR laboratory OR inflammatory OR inflammation OR coagulation OR CRP
 OR "C-reactive protein" OR procalcitonin OR leukocyte* OR platelet*
 OR fibrinogen OR INR OR aPTT OR D-dimer* OR albumin OR hemoglobin)
```

S1.4 Stream B (histological) — PubMed/MEDLINE — 80 records retrieved, 1 retained

```
("NexoBrid"[Title/Abstract] OR Nexobrid[Title/Abstract]
 OR "bromelain-based enzymatic debridement"[Title/Abstract]
 OR "enzymatic debridement"[Title/Abstract]
 OR "enzymatic necrosectomy"[Title/Abstract])
AND
(burn*[Title/Abstract] OR "thermal injury"[Title/Abstract]
 OR "burn wound"[Title/Abstract])
AND
(clinical[Title/Abstract] OR outcome*[Title/Abstract] OR healing[Title/Abstract]
 OR graft*[Title/Abstract] OR epithelialization[Title/Abstract]
 OR infection[Title/Abstract] OR scar*[Title/Abstract]
 OR histolog*[Title/Abstract] OR histopatholog*[Title/Abstract]
 OR biopsy[Title/Abstract] OR immunohistochem*[Title/Abstract]
 OR immunostain*[Title/Abstract] OR "tissue remodeling"[Title/Abstract]
 OR remodeling[Title/Abstract] OR collagen[Title/Abstract]
 OR elastin[Title/Abstract] OR "extracellular matrix"[Title/Abstract]
 OR angiogenesis[Title/Abstract] OR microvascular*[Title/Abstract]
 OR vascular*[Title/Abstract] OR CD31[Title/Abstract] OR CD34[Title/Abstract]
 OR VEGF[Title/Abstract] OR Ki-67[Title/Abstract] OR Ki67[Title/Abstract]
 OR MMP*[Title/Abstract] OR pseudoeschar[Title/Abstract])
```

S1.5 Stream B (histological) — Embase — 118 records retrieved, 2 retained

```
('nexobrid'/exp OR nexobrid:ti,ab
 OR 'bromelain based enzymatic debridement':ti,ab
 OR 'enzymatic debridement':ti,ab
 OR 'enzymatic necrosectomy':ti,ab)
AND
('burn'/exp OR burn*:ti,ab OR 'thermal injury':ti,ab OR 'burn wound':ti,ab)
AND
(clinical:ti,ab OR outcome*:ti,ab OR healing:ti,ab OR graft*:ti,ab
 OR epithelialization:ti,ab OR infection:ti,ab OR scar*:ti,ab
 OR histolog*:ti,ab OR histopatholog*:ti,ab OR biopsy:ti,ab
 OR immunohistochem*:ti,ab OR immunostain*:ti,ab
 OR 'tissue remodeling':ti,ab OR collagen:ti,ab OR elastin:ti,ab
 OR 'extracellular matrix':ti,ab OR angiogenesis:ti,ab
 OR microvascular*:ti,ab OR vascular*:ti,ab OR CD31:ti,ab OR CD34:ti,ab
 OR VEGF:ti,ab OR Ki67:ti,ab OR MMP*:ti,ab OR pseudoeschar:ti,ab)
```

S1.6 Stream B (histological) — Scopus — 102 records retrieved, 2 retained

```
TITLE-ABS(NexoBrid OR "bromelain-based enzymatic debridement"
 OR "enzymatic debridement" OR "enzymatic necrosectomy")
AND
TITLE-ABS(burn* OR "thermal injury" OR "burn wound")
AND
TITLE-ABS(clinical OR outcome* OR healing OR graft* OR epithelialization
 OR infection OR scar* OR histolog* OR histopatholog* OR biopsy
 OR immunohistochem* OR immunostain* OR "tissue remodeling" OR collagen
 OR elastin OR "extracellular matrix" OR angiogenesis OR microvascular*
 OR vascular* OR CD31 OR CD34 OR VEGF OR Ki67 OR MMP* OR pseudoeschar)
```

Table S2. Reports excluded at the full-text stage, with reasons

Report	Reason for exclusion	Use in the review
**Mataro 2017 [40]**	No eligible outcome reported: clinical evolution and scar scores only, with no systemic biological parameter and no histopathology; meets the criteria of neither stream	Not used in the synthesis
**Sharaf 2024 [34]**	Study design not eligible: in vitro laboratory study	Retained as supporting evidence and cited in the Discussion
**Vanaclocha 2024 [41]**	Population and intervention not eligible: the cohort is not defined by treatment with bromelain-based enzymatic debridement	Not used
**Borges 2026 [42]**	Population and intervention not eligible: bromelain-based enzymatic debridement is not the studied intervention in an eligible population	Not used

Reports excluded at the full-text stage are listed in accordance with PRISMA item 16b; their full citations are given in the reference list of the main text. Preclinical (porcine and murine) studies retrieved by the searches were excluded from the synthesis by the design criterion and are not listed individually here; those cited as supporting evidence appear in the reference list of the main text.

Table S3. Biological parameters measured and sampling schedule (Stream A)

Study	Domain	Analytes / methods	Sampling time points
**Martín 2018 [26]**	Coagulation, haematology	Haemoglobin, platelets, prothrombin time (% and ratio), aPTT ratio, fibrinogen	Pre-application; 24 h post-debridement; after phytomenadione; 48 h; 72 h
**Hofmaenner 2021 [18]**	Inflammation, biochemistry, haemodynamics	Leukocyte, neutrophil and lymphocyte counts, CRP, procalcitonin, lactate, central venous oxygen saturation; sodium, potassium, chloride, magnesium, phosphate, calcium; creatinine and eGFR; blood pressure, heart rate, body temperature, catecholamine requirement, opioid and anaesthetic dosage. Bleeding defined a priori as a haemoglobin fall >1 g/dl with clinical signs of blood loss	Four time points: −24 h, +24 h, +24–48 h, +48–72 h relative to debridement
**Sharaf 2022 [17]**	Microbiology	Microbiological surface wound swabs, with new emergence of organisms recorded before and after debridement and classified by Gram stain and species	On initial assessment, at the time of debridement, and weekly thereafter per unit protocol; followed to healing (mean 6 weeks in the ED group)
**Hasham 2023 [27]**	Coagulation, haematology	PT, aPTT, thrombin time, haemoglobin, haematocrit	Case 1: admission, day 1 pre-ED, day 1 post-ED, day 8, day 55, day 65. Case 2: admission only (values not reported)
**Pfister 2023 [14]**	Coagulation; inflammation; biochemistry	Primary: INR, aPTT, fibrinogen, factor V, factor XIII, platelet count, and the substitution of erythrocytes, platelets, fresh frozen plasma, fibrinogen concentrate, factor XIII concentrate and prothrombin complex concentrate. Secondary: lactate, pH, calcium, haemoglobin, haematocrit, leukocytes, CRP, procalcitonin, bilirubin, AST, ALT, GGT; body temperature and fluid administration. The most comprehensive panel in the review	Daily, day 1 to day 7 of hospitalisation
**Capitelli-McMahon 2024 [28]**	Coagulation	Prothrombin time (local range 9.5–12.5 s), aPTT (22–32 s), platelet count (135–400 g/l)	Day −1, day 0 (day of debridement) and days 1, 2 and 3
**Deplazes 2024 [16]**	Microbiology	Blood cultures, drawn on clinical indication; coagulase-negative staphylococci and Cutibacterium required ≥2 positive cultures from the same episode to count	Continuously throughout the ICU stay; no protocolised schedule
**Breidung 2025 [15]**	Inflammation, coagulation, haematology, biochemistry	Leukocyte count, haemoglobin, prothrombin ratio (Quick, % of normal), aPTT, sodium, potassium, creatinine, CRP; plus body temperature and pain (NRS 0–10). Platelets, INR, fibrinogen, D-dimer, albumin, procalcitonin, lactate and liver enzymes were not measured	Two time points only: within 24 h before and within 24 h after application
**Bulla 2025 [11]**	Coagulation; fever; infection	Prothrombin time and aPTT in all patients before debridement and, where performed, afterwards; blood transfusion requirement; early post-ED fever; clinical infection with pathogen identification	Before debridement in all 300 patients; after debridement in 226 (no post-treatment coagulation test was performed in 74). Fever and infection recorded throughout admission
**Kaita 2025 [29]**	Microbiology, transfusion	Blood cultures (bacteraemia); transfused volumes of red cells, fresh frozen plasma and platelet concentrate; intratissue (compartment) pressure in two cases. No blood biochemistry or haematology of any kind was measured	Not specified for cultures; compartment pressure before and 4 h after application

No two studies measured the same set of analytes at the same time points. The panels range from three parameters at five time points (Capitelli-McMahon [28]) to more than twenty parameters daily for a week (Pfister [14]), and two studies measured no blood analyte at all (Kaita [29]; and Miura [20] in Stream B). This heterogeneity is the structural reason why quantitative pooling was not possible.

Table S4. Residual data gaps in the included studies after full-text retrieval

Study	Status	Remaining gap
**Di Lonardo 2018 [19]**	Full text obtained	No quantitative measurement exists in the paper; no funding or conflict-of-interest statement is provided
**Martín 2018 [26]**	Full text obtained	None
**Hofmaenner 2021 [18]**	Full text obtained	Per-analyte raw values are presented only in figures; the numerical table reports predicted means, differences and adjusted *p*-values, from which absolute values at each time point cannot be recovered
**Sharaf 2022 [17]**	Full text obtained	No statistical analysis exists to extract. Organism frequencies are complete and tabulated by week, but no comparison between cohorts was tested, so no effect estimate can be derived without reanalysis of individual data
**Hasham 2023 [27]**	Full text obtained	None
**Pfister 2023 [14]**	Full text obtained	None material. Coagulation-factor values are tabulated in full; bleeding events are not separately reported, only transfusion volumes
**Capitelli-McMahon 2024 [28]**	Full text obtained	No clinical outcome of any kind is reported — not grafting, wound closure, length of stay or mortality. This is a feature of the study design, not a retrieval failure. Numeric laboratory values appear only in box plots, with means given in the text
**Deplazes 2024 [16]**	Full text obtained	No length of stay, ventilation duration, grafting rate or wound-closure time; no inflammatory or biochemical analyte
**Breidung 2025 [15]**	Full text obtained	Exact pre/post values for aPTT, sodium, potassium and creatinine are not tabulated, only reported as non-significant
**Bulla 2025 [11]**	Full text obtained	Post-debridement coagulation testing was not performed in 74 of 300 patients, and the scar hypertrophy field is incomplete in 99 of 300. Neither gap can be filled retrospectively
**Kaita 2025 [29]**	Full text obtained	No laboratory analyte was measured; nothing to retrieve
**Miura 2025 [20]**	Full text obtained	Fixation protocol not stated. All other items are reported

All twelve full texts were obtained and read. The gaps that remain are gaps in the published studies themselves, not gaps in retrieval — a distinction that matters for the interpretation of the synthesis.

Table S5. PRISMA 2020 checklist

Section and Topic	Item #	Checklist item	Location where item is reported
**TITLE**			
Title	1	Identify the report as a systematic review.	Title page
**ABSTRACT**			
Abstract	2	See the PRISMA 2020 for Abstracts checklist.	Abstract
**INTRODUCTION**			
Rationale	3	Describe the rationale for the review in the context of existing knowledge.	Introduction, 1.1–1.6
Objectives	4	Provide an explicit statement of the objective(s) or question(s) the review addresses.	1.7
**METHODS**			
Eligibility criteria	5	Specify the inclusion and exclusion criteria for the review and how studies were grouped for the syntheses.	2.3; Table 1
Information sources	6	Specify all databases, registers, websites, organisations, reference lists and other sources searched or consulted to identify studies. Specify the date when each source was last searched or consulted.	2.2.1
Search strategy	7	Present the full search strategies for all databases, registers and websites, including any filters and limits used.	2.2.1; Suppl. Table S1
Selection process	8	Specify the methods used to decide whether a study met the inclusion criteria of the review, including how many reviewers screened each record and each report retrieved, whether they worked independently, and if applicable, details of automation tools used in the process.	2.4
Data collection process	9	Specify the methods used to collect data from reports, including how many reviewers collected data from each report, whether they worked independently, any processes for obtaining or confirming data from study investigators, and if applicable, details of automation tools used in the process.	2.5
Data items	10a	List and define all outcomes for which data were sought. Specify whether all results that were compatible with each outcome domain in each study were sought (e.g. for all measures, time points, analyses), and if not, the methods used to decide which results to collect.	2.5; 2.7
	10b	List and define all other variables for which data were sought (e.g. participant and intervention characteristics, funding sources). Describe any assumptions made about any missing or unclear information.	2.5
Study risk of bias assessment	11	Specify the methods used to assess risk of bias in the included studies, including details of the tool(s) used, how many reviewers assessed each study and whether they worked independently, and if applicable, details of automation tools used in the process.	2.6
Effect measures	12	Specify for each outcome the effect measure(s) (e.g. risk ratio, mean difference) used in the synthesis or presentation of results.	2.7
Synthesis methods	13a	Describe the processes used to decide which studies were eligible for each synthesis (e.g. tabulating the study intervention characteristics and comparing against the planned groups for each synthesis (item #5)).	2.3; 2.7
	13b	Describe any methods required to prepare the data for presentation or synthesis, such as handling of missing summary statistics, or data conversions.	2.5; 2.7
	13c	Describe any methods used to tabulate or visually display results of individual studies and syntheses.	2.7; Tables 2–9; Figure 1

Section and Topic	Item #	Checklist item	Location where item is reported
	13d	Describe any methods used to synthesize results and provide a rationale for the choice(s). If meta-analysis was performed, describe the model(s), method(s) to identify the presence and extent of statistical heterogeneity, and software package(s) used.	2.7
	13e	Describe any methods used to explore possible causes of heterogeneity among study results (e.g. subgroup analysis, meta-regression).	N/A; see 2.7, 3.3, Suppl. Table S3
	13f	Describe any sensitivity analyses conducted to assess robustness of the synthesized results.	3.5
Reporting bias assessment	14	Describe any methods used to assess risk of bias due to missing results in a synthesis (arising from reporting biases).	Not assessed; Section 5
Certainty assessment	15	Describe any methods used to assess certainty (or confidence) in the body of evidence for an outcome.	Not assessed; Sections 2.7 and 5
**RESULTS**			
Study selection	16a	Describe the results of the search and selection process, from the number of records identified in the search to the number of studies included in the review, ideally using a flow diagram.	3.1; Figure 1
	16b	Cite studies that might appear to meet the inclusion criteria, but which were excluded, and explain why they were excluded.	3.1; Suppl. Table S2
Study characteristics	17	Cite each included study and present its characteristics.	3.2; Table 2
Risk of bias in studies	18	Present assessments of risk of bias for each included study.	3.5; Table 8
Results of individual studies	19	For all outcomes, present, for each study: (a) summary statistics for each group (where appropriate) and (b) an effect estimate and its precision (e.g. confidence/credible interval), ideally using structured tables or plots.	3.3, 3.4; Tables 3–7
Results of syntheses	20a	For each synthesis, briefly summarise the characteristics and risk of bias among contributing studies.	3.2, 3.5
	20b	Present results of all statistical syntheses conducted. If meta-analysis was done, present for each the summary estimate and its precision (e.g. confidence/credible interval) and measures of statistical heterogeneity. If comparing groups, describe the direction of the effect.	N/A; see 3.3, 3.4, 3.6
	20c	Present results of all investigations of possible causes of heterogeneity among study results.	3.3, 3.5, 3.6
	20d	Present results of all sensitivity analyses conducted to assess the robustness of the synthesized results.	3.5
Reporting biases	21	Present assessments of risk of bias due to missing results (arising from reporting biases) for each synthesis assessed.	Not assessed; Section 5
Certainty of evidence	22	Present assessments of certainty (or confidence) in the body of evidence for each outcome assessed.	Not assessed; Sections 2.7 and 5
**DISCUSSION**			
Discussion	23a	Provide a general interpretation of the results in the context of other evidence.	4.1–4.4, 4.6
	23b	Discuss any limitations of the evidence included in the review.	3.5; 4.1–4.3; Section 5
	23c	Discuss any limitations of the review processes used.	Section 5

Section and Topic	Item #	Checklist item	Location where item is reported
	23d	Discuss implications of the results for practice, policy, and future research.	4.5; Section 6
**OTHER INFORMATION**			
Registration and protocol	24a	Provide registration information for the review, including register name and registration number, or state that the review was not registered.	2.1; PROSPERO CRD420261492222
	24b	Indicate where the review protocol can be accessed, or state that a protocol was not prepared.	2.1; PROSPERO record
	24c	Describe and explain any amendments to information provided at registration or in the protocol.	2.2.2
Support	25	Describe sources of financial or non-financial support for the review, and the role of the funders or sponsors in the review.	Funding
Competing interests	26	Declare any competing interests of review authors.	Conflicts of Interest
Availability of data, code and other materials	27	Report which of the following are publicly available and where they can be found: template data collection forms; data extracted from included studies; data used for all analyses; analytic code; any other materials used in the review.	Data Availability; Suppl. Materials

Table S6. SWiM reporting checklist

Item	Reporting item	Location and content
**1**	Grouping studies for synthesis — describe how studies were grouped and why	2.3, 2.7 — grouped by evidence stream and, within each stream, by outcome domain: coagulation and haemostasis; infection, bacteraemia and microbiological profile; inflammatory and biochemical markers; clinical outcomes and safety. Groupings were defined before data extraction
**2**	Describe the standardized metric and the rationale	2.7 — no standardized metric was used; effect estimates are reported as given by each study, in their original units, and are never converted or pooled
**3**	Describe the synthesis method	2.7 — narrative synthesis. Studies are presented within each outcome group in order of decreasing methodological robustness. Vote counting based on statistical significance was avoided
**4**	Criteria used to prioritize results for summary and synthesis	2.7, 3.3 — priority was given to studies with a comparator, to adjusted effect estimates and to prospectively defined outcomes; case-level reports are presented separately and never as equivalent to cohort data
**5**	Investigation of heterogeneity in reported effects	3.3, 3.5, 3.6 and Table S3 — heterogeneity in analytes, sampling schedules, comparators and outcome definitions is described narratively and is the stated reason why no meta-analysis was performed. A narrative sensitivity assessment, reported in 3.5, states which findings persist when the study at critical risk of bias and the four at serious risk are removed
**6**	Certainty of evidence — describe the methods used to assess certainty	Not assessed. The reasons for not applying a structured certainty framework are given in 2.7, and the omission is retained as a limitation in Section 5
**7**	Data presentation methods	2.7; Tables 2–9; Figure 1 — structured tabulation by outcome domain, with a cross-tabulation of outcome domains by study as the primary endpoint (Table 9)
**8**	Reporting results — describe the synthesis findings and their certainty	3.3, 3.4, 3.6; sensitivity assessment in 3.5. No certainty rating accompanies the findings; see 2.7 and Section 5
**9**	Limitations of the synthesis	Section 5
